# Artificial Intelligence for Tumor Tissue Detection in Stomach Cancer: A Retrospective Algorithm Development and Validation Study

**DOI:** 10.3390/jcm15093370

**Published:** 2026-04-28

**Authors:** Nikolay Karnaukhov, Vincenzo Davide Palumbo, Mark Voloshin, Alexander Mongolin, Alexander Skvortsov, Ainur Karimov, Yuri Gorbachev, Konstantin Abramov, Anastasia Zabruntseva, Georgy Yakubovsky, Aleksandra Asaturova, Andrea Palicelli, Sergey Khomeriki, Igor Khatkov

**Affiliations:** 1 A.S. Loginov Moscow Clinical Scientific Center, 111123 Moscow, Russiamarkvoloshin95@gmail.com (M.V.); y.v.gorbachev@mail.ru (Y.G.); konstantin.abramov.1994@mail.ru (K.A.); anastasyazabruntseva@gmail.com (A.Z.); yakugeo@gmail.com (G.Y.); i.hatkov@mknc.ru (I.K.); 2 Institute of Clinical Morphology and Digital Pathology, Sechenov University, 119991 Moscow, Russia; 3 Triolo-Zancla Hospital, 90133 Palermo, Italy; vincenzopalumbo84@libero.it; 4 Institute of Artificial Intelligence, 420500 Innopolis, Russia; d20180761@novaims.unl.pt (A.M.); skvorec347680@gmail.com (A.S.); ainureg@yandex.ru (A.K.); 5 Nova Information Management School, Universidade Nova de Lisboa, Campus de Campolide, 1070-312 Lisboa, Portugal; 6 National Medical Research Center for Obstetrics, Gynecology and Perinatology Named After Academician V.I. Kulakov, Ministry of Health of Russia, 117513 Moscow, Russia; a.asaturova@gmail.com; 7 Department of Pathological Anatomy and Clinical Pathological Anatomy, Institute of Human Biology and Pathology, Pirogov Russian National Research Medical University, 117997 Moscow, Russia; 8 Pathology Unit, Azienda USL—IRCCS di Reggio Emilia, 42123 Reggio Emilia, Italy

**Keywords:** artificial intelligence (AI), artificial neural networks (ANNs), convolutional neural networks (CNNs), stomach cancer

## Abstract

**Background:** Gastric cancer remains one of the leading causes of cancer-related mortality worldwide, underscoring the need for more effective diagnostic strategies. This study aims to use annotated digitized histological slides of gastric cancer and precancerous lesions to develop artificial intelligence algorithms for the diagnosis of gastric lesions. **Materials and Methods:** We developed a deep learning tool using a training cohort of 970 digitized gastric biopsy slides. Convolutional neural networks (CNNs) were trained for histological recognition and ICD-10 code assignment. The model was validated on an independent test cohort of 250 cases, with expert consensus as the reference standard. Performance was assessed using sensitivity, specificity, and Cohen’s kappa. Survival analysis used Kaplan–Meier, log-rank tests (SPSS 16.0; *p* < 0.05 significant). **Results:** Analysis of the training cohort led to a scoring system predicting fatal outcomes based on age and morphology (high-grade component > 70%, ulceration, absence of metaplasia/dysplasia). High-risk patients (4–5 points) had significantly worse survival than low-risk patients (0–3 points) (Log Rank = 14,754; *p* < 0.0001). One-year survival was 71% (low-risk) vs. 40% (high-risk); mean survival was 19.2 vs. 11.3 months. In the test cohort, the AI algorithm demonstrated 79.6% sensitivity and 86.7% specificity (*p* < 0.0001) for differentiating malignant from benign gastric lesions. **Conclusions:** A system combining AI-based analysis with a prognostic scoring model has been developed to reduce diagnostic errors and improve risk stratification in gastric cancer pathology.

## 1. Introduction

Oncological diseases rank among the leading causes of morbidity and mortality worldwide. According to global statistics, the incidence of gastric malignancies is 6.9 per 100,000 population [1]. Due in large part to advances in endoscopic technologies, gastric tumors are being diagnosed with increasing frequency worldwide [1].

In parallel with the rising cancer incidence, the number of in vivo pathological examinations has also increased. The multistage nature of this process, its complexity, and the involvement of a large number of specialists have necessitated the introduction of laboratory information systems. Furthermore, the advent of equipment for digitizing histological slides has given rise to a new field in pathology—digital pathology.

The accumulation of digital archives and growing expertise in digital pathology are opening new opportunities for the integration of artificial intelligence (AI) systems. Accordingly, refining approaches in morphological diagnostics to improve accuracy and reduce turnaround time has become a pressing challenge, while the shortage of qualified personnel underscores the need for innovative solutions. In this context, training pathologists to make effective use of AI tools and to critically evaluate AI-assisted results is of primary importance [2].

Machine learning, a major branch of AI, employs mathematical and statistical methods to enhance computational performance [3]. Deep learning represents a subset of machine learning characterized by the use of multilayered artificial neural networks (ANNs). The term “deep learning” encompasses a group of novel methods that have demonstrated substantial performance improvements over state-of-the-art machine learning algorithms across multiple disciplines [4]. These methods have revolutionized image classification and speech recognition due to their flexibility and high accuracy [5]. Such breakthroughs have led to the adoption of deep learning as an effective approach for solving diverse problems in medicine. In disease diagnostics, the application of deep learning to radiological and histological image classification has, in several instances, achieved performance comparable to that of human experts [6].

Various types and architectures of ANNs are being explored in pathology, with convolutional neural networks (CNNs) being the most widely applied. CNNs represent a subtype of ANNs that reduce the dimensionality of input data by applying convolutional filters presumed to capture relevant features. They were specifically designed for applications involving automated image recognition. Among recurrent neural networks, the most common architecture is the Hopfield network [7]. This fully connected ANN links the output of each neuron to all others, while each neuron also has its own input, enabling the solution of combinatorial optimization tasks (e.g., restoration of damaged images). Their training relies on specially designed as well as hybrid algorithms.

For example, B. Ehteshami Bejnordi et al. developed an algorithm for the detection of breast cancer metastases in lymph nodes. Achieving an accuracy of 96% in identifying both macro- and micrometastases, the authors proposed using their algorithm as an AI-based prescreening tool to accelerate histological evaluation. In this workflow, the system “highlights” potential metastatic foci, which are then confirmed or rejected by the pathologist [6]. Similarly, Z. Alom et al. trained a neural network to discriminate between benign and malignant tumors with an accuracy of 97% [8].

Comparisons of convolutional neural network (CNN)-based analysis of histological images with assessments by experienced pathologists demonstrated that, in the absence of time constraints, pathologists consistently achieved the highest accuracy. However, when time limitations were imposed, diagnostic accuracy decreased in human experts, whereas the performance of CNNs remained stable.

The most promising results have been reported when AI is integrated as a supportive tool in the diagnostic process. For instance, Steiner D.F. et al. showed that the accuracy of detecting micrometastases in lymph nodes increased to 91.2% when pathologists collaborated with AI, compared with 83.3% when either pathologists or neural networks worked independently [9]. Although the majority of publications focus on oncological pathology, there are also reports on training neural networks to detect celiac disease, ulcerative colitis, gastritis, or to perform automated cell counting for the diagnosis of autoimmune hepatitis [10,11,12,13].

The ethical implications of AI in pathology are widely debated in the literature. It is envisioned—perhaps soon to become reality—that AI technologies in pathology will optimize and improve various stages of diagnostic workflows. However, if implemented without proper legal and social safeguards, AI may compromise the deontological principles of medical practice. Chauhan C. et al. published a comprehensive review addressing the ethical aspects of AI in pathology [14]. They highlighted three core principles for the ethical use of AI in this field: transparency, accountability, and governance. The pathology of the future must be guided by these principles. Pathologists should be aware of AI capabilities to deliver improved patient care while remaining attentive to associated ethical challenges. Crucially, it is pathologists themselves who must be actively engaged in the development, training, and clinical implementation of diagnostic algorithms to ensure their responsible and effective integration into pathology practice.

The aim of this study is to develop an artificial intelligence (AI) algorithm capable of recognizing various histological structures in digitized histological slides and assigning gastric lesion codes according to the International Classification of Diseases (ICD-10). In addition, during the annotation of histological slides of gastric cancer and precancerous gastric mucosal lesions for AI training, precise quantitative measures of histological types will be obtained. These data are intended to be used for histological reclassification and for revising clinically significant threshold values based on survival outcomes. Thus, in addition to AI development, the study will explore the potential for predicting patient outcomes in verified gastric carcinoma through statistical analysis of annotated histological data.

## 2. Materials and Methods

The study was conducted at the Pathology Department of the Moscow Clinical Scientific Center named after Loginov. At the first stage, a training dataset was assembled, followed by AI algorithm training for the recognition of histological structures and the development of an algorithm for ICD-10 code assignment based on detected epithelial formations in digitized gastric biopsy specimens.

The training dataset consisted of biopsy material obtained during endoscopic gastric examinations from 970 patients, including 472 cases of malignant epithelial tumors, 40 cases of high-grade dysplasia, 42 cases of low-grade dysplasia, and 416 cases of non-neoplastic pathology. To ensure consistency in annotation, scanning, and subsequent computational analysis, only cases in which all fragments from an endoscopic procedure were embedded in a single paraffin block were included. Thus, each patient contributed one histological slide containing between one and five fragments.

Histological slides were prepared using standard protocols, stained with hematoxylin and eosin, and digitized with a Leica GT 450 DX whole-slide scanner (Leica Biosystems Nussloch GmbH, Nussloch, Germany). Annotation of histological structures was performed using criteria based on the World Health Organization (WHO) classification principles.

**Figure 1:**
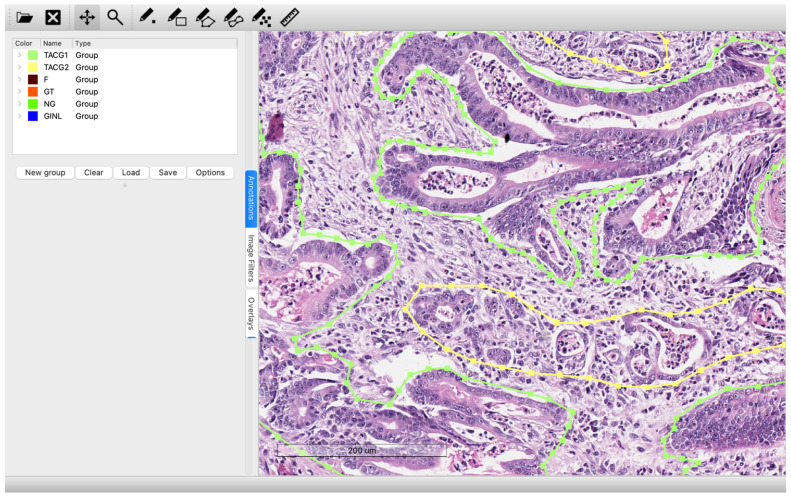
An example of annotating a scanned histological specimen with a list of all marked classes in the scan using the ASAP software.

Each digitized histological specimen was stored as a separate .svs file with a corresponding annotation file in .asap format. Annotated regions were subdivided into smaller image slices of 256 × 256 pixels. Each slice was classified by the neural network according to localization-based class tables. Figure 1 shows an example of marking a scanned histological specimen with a list of all marked classes in the scan using the ASAP software version 2.2 (ASAP project team, Universidad Politécnica de Madrid, Spain).

Annotated images were used to train convolutional neural networks (CNNs) designed for feature extraction and interpretation. The developed system consisted of a three-level ensemble. The first-level network was trained to classify each slice as containing cancer, other pathologies, or normal tissue. Second-level networks were trained to classify slices containing cancer, other pathologies, or normal tissue, respectively, by morphological type (see Supplementary Materials Table S1). The final-level network assigned an ICD-10 code based on prediction statistics for all slices in a single scan. A known issue was that, according to diagnostic rules, the presence of even a single slice containing a tumor should result in the assignment of the final code C16.9. To avoid a high rate of false-positive results, isolated, discrete slices classified as tumors were treated as outliers and ignored by the final-level network when determining the ICD-10 code. This represents a limitation of the current CNNs for diagnosing biopsy specimens containing only minimal amounts of tumor (e.g., small clusters of cancer cells or isolated tumor structures). The resulting slice-by-slice classification was interpreted as a high-resolution image segmentation. For each case, the output included an .xml file for visualization in ASAP (allowing comparison across classes) and a thumbnail image for preliminary inspection.

During training, data augmentation techniques and stain normalization were applied, which improved model stability against color variability in source images. For model preparation, the dataset of segmented images was divided into training and validation cohorts at a 4:1 ratio. Model performance was assessed using confusion matrix analysis for both training and validation sets.

The second stage of the study involved the analysis of the trained AI algorithm’s performance, a comprehensive re-evaluation of all histological slides by an expert panel, and a comparison of expert pathologists’ assessments with the algorithm’s image analysis results.

For this purpose, a test dataset was compiled from gastric biopsy material obtained during endoscopic examinations of 250 patients, including 100 cases of malignant epithelial tumors, 25 cases of high-grade dysplasia, 25 cases of low-grade dysplasia, and 100 cases of non-neoplastic pathology. The test dataset was independent of the training dataset.

**Figure 2:**
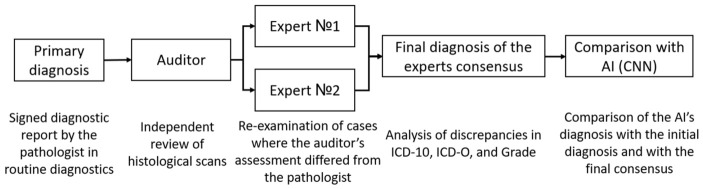
Scheme of validation of test dataset for subsequent verification of accuracy of AI work.

Each whole-slide image in the test set was initially reviewed by an auditor. Based on this audit and comparison with the original diagnostic report, selected scans were subsequently reviewed independently by two experts, after which the panel assigned ICD-10 and ICD-O codes (Figure 2).

Statistical analyses were performed across all parameters to determine the sensitivity and specificity of the models for each localization, expressed in terms of ICD-10 code assignment. All statistical analyses were conducted using SPSS 16.0 for Windows (SPSS Inc., Chicago, IL, USA). All reported *p*-values were two-sided, with *p* < 0.05 considered statistically significant. Quantitative data are presented as mean ± standard deviation. Frequencies of categorical variables were compared using the chi-square (χ^2^) test, with Yates’ correction applied when appropriate.

Algorithm performance was further evaluated using confusion matrix analysis, with calculation of class prediction probabilities. Kaplan–Meier survival analysis was employed to construct survival curves and estimate cumulative survival rates and mean survival times. Differences between groups were assessed with the log-rank test. Interobserver agreement among experts was analyzed using Cohen’s kappa coefficient, along with extraction of sensitivity and specificity values.

## 3. Results

All endoscopic gastric biopsies included in the training dataset were obtained from patients with a predominance of females (male-to-female ratio = 0.7). The mean age across all groups was 60 years (61 years for men, 59 years for women). The minimum age was 18 years for women and 20 years for men, while the maximum was 93 and 88 years, respectively.

Biopsies from gastric tumors of all anatomical sites were included in the study. However, the most common tumor localizations were the pyloric antrum (C16.3, 40%) and the gastric body (C16.2, 27%). In 11% of cases, based on endoscopic findings, tumors extended across multiple gastric regions (C16.8).

Patients with non-neoplastic gastric pathology had been under gastroenterological observation for clinical manifestations of chronic gastritis (e.g., epigastric pain, postprandial heaviness). In several cases, follow-up with repeat endoscopic evaluation and biopsy after anti-inflammatory therapy was performed; however, no malignant growth was detected in any of these patients during the observation period.

In slightly more than half of the patients (*n* = 244, 52%), TNM staging was established clinically based on endoscopic examination, endoscopic ultrasound, computed tomography, and other imaging modalities. Surgical treatment was performed in 228 patients (48%), including submucosal resection (58 patients, 12%), distal or proximal gastrectomy (122 patients, 25%), and total gastrectomy (48 patients, 10%).

After digitization and annotation of the slides, annotated regions were subdivided into pixels, which were further grouped into square image patches, hereafter referred to as “slices.” All slices were organized into class-specific folders. The total biopsy area amounted to 84,075,821,598 pixels, of which 76,321,799,162 pixels (90% of the biopsy area) were annotated.

All slices were reviewed, and those containing more than 10% of unannotated areas or annotations inconsistent with the corresponding histological features were excluded from the study.

The most frequent categories were non-neoplastic classes, comprising 52,490,089,929 pixels. For the first time, it was quantified that gastric carcinoma biopsies contained up to 13,924,331,585 pixels of non-neoplastic tissue. In other words, approximately 35.3% of the tissue area in carcinoma biopsies consisted of non-neoplastic components. Dysplastic changes (glandular intraepithelial neoplasia, low- and high-grade) accounted for 1,175,304,982 pixels (2.98%), while the remaining 61.7% corresponded to carcinomas of various histological subtypes. These findings may be of practical relevance for selecting tissue prior to molecular genetic testing, as the proportion of tumor tissue directly affects the quality of molecular diagnostic results. According to published data, a minimum of 200 tumor cells and a tumor-to-nontumor ratio exceeding 20% are required for reliable molecular testing [15].

Tumor tissue was annotated across 20,709,875,415 pixels, high-grade dysplasia across 620,274,997 pixels, and low-grade dysplasia across 2,501,558,821 pixels. Among tumor classes, the most frequently annotated subtype was tubular adenocarcinoma, low-grade (“TAC-LG,” 6,664,074,401 pixels), followed by tubular adenocarcinoma, high-grade (“TAC-HG,” 4,598,290,866 pixels), poorly cohesive carcinoma, signet-ring cell subtype (“PCC,” 4,244,555,130 pixels), poorly cohesive carcinoma, NOS (“PCC-NOS,” 2,510,671,578 pixels), and papillary adenocarcinoma, low-grade (“PAC-LG,” 1,817,583,758 pixels). Less frequently annotated were mucinous adenocarcinomas (“MAC,” 543,032,134 pixels), neuroendocrine carcinoma structures (“NED,” 144,753,471 pixels), undifferentiated carcinomas (“NDC,” 101,471,957 pixels), adenocarcinomas with lymphoid stroma (“ACLS,” 69,131,679 pixels), and micropapillary adenocarcinomas (“MPAC,” 16,310,441 pixels).

For each digitized slide, the precise pixel counts for all histological structures were recorded. To assign one of the four ICD-10 codes included in the study, formulas were developed linking annotated histological structures to ICD-10 classification.

Detection of features consistent with non-neoplastic pathology was designated as “K29.7.” Further subclassification by gastritis type or ulcerative changes in the gastric mucosa was beyond the scope of this study; the primary objective was to exclude evidence of tumor growth within the examined material. Detection of glandular intraepithelial neoplasia, high-grade (GINH), in the absence of invasive carcinoma was designated as “D00.2,” whereas detection of glandular intraepithelial neoplasia, low-grade (GINL), without evidence of invasive or non-invasive carcinoma, was designated as “D13.1.”

The identification of histological structures characteristic of gastric carcinomas according to the WHO nomenclature, designated in the developed reference guide as TACG1, TACG2, TACG3, PACG1, PACG2, MPAC, PCC, PCC-NOS, MAC1, MAC2, ACLS, HAC, ACFG, SCC, NDC, and NED, was classified as “C16.9.”

Across the processed slices, non-neoplastic tissue (K29.7) predominated in all scans, as it is invariably present in all nosological categories, and was identified in 52,490,089,929 pixels. Invasive carcinoma accounted for 20,709,875,415 pixels, carcinoma in situ for 620,274,997 pixels, and low-grade dysplastic structures for 2,501,558,821 pixels.

Tumor-related classes represented 29.7% of all annotated histological structures (*n* = 721), while low-grade dysplasia accounted for 5.4% (*n* = 132), high-grade dysplasia for 3.3% (*n* = 80), and the remaining 61.6% (*n* = 1494) corresponded to non-neoplastic classes.

In total, 76,321,799,162 pixels were annotated. A further 7,754,022,436 pixels remained unannotated, encompassing artifacts of histological slide preparation (minor folds), artificial alterations (fragmented glands), stromal elements, and areas with indeterminate pathological changes that may accompany, but are not directly part of, tumor growth.

Accurate annotation of all histological structures contributing to the determination of the histological subtype of gastric carcinoma according to the WHO classification allows for a more precise definition of tumor subtypes and reduces subjectivity in assessing the relative proportions of individual components. To establish the exact subtype from annotated digitized histological images, formulas were developed enabling the determination of the histological type according to WHO criteria by combining various annotated structural patterns.

Within the group of adenocarcinomas NOS, 100% of cases were further subtyped. Isolated cases where grade was not indicated were reassigned to high-grade tubular adenocarcinoma. A total of 42.1% of low-grade adenocarcinomas NOS were reclassified as tubular adenocarcinomas low-grade, a shift that may be regarded as a minor adjustment. However, the reclassification of low-grade adenocarcinomas NOS into high-grade tubular adenocarcinomas likely represents a more serious diagnostic oversight. This occurred in slightly over one-quarter of cases (26.3%). It remains necessary to further test the hypothesis that the selection of high-grade malignancy status depends on the proportion of high-grade component within the tumor. In these cases, the high-grade component occupied only a minor proportion of the tumor area, which likely contributed to undergrading during the initial diagnostic assessment.

Additionally, 15.8% of low-grade adenocarcinomas NOS were reclassified as papillary adenocarcinomas low-grade, 10.5% as mucinous adenocarcinomas, and the remaining cases as adenocarcinomas with mixed subtypes.

After reclassification based on the annotated data, 60% of cases initially diagnosed as high-grade adenocarcinomas NOS were reassigned to tubular adenocarcinomas, high-grade. Notably, 35% of cases in this group were reclassified as poorly cohesive carcinomas (25% signet-ring cell subtype and 10% non-signet-ring subtype), while the remaining 5% were designated as adenocarcinomas with mixed subtypes.

Primary tubular adenocarcinomas, low-grade, retained their original classification in more than half of cases (59%). The major trend observed was an upgrading of malignancy grade to high-grade in 30.8% of cases. Approximately 5% of tumors were reclassified into papillary adenocarcinomas (some of them with grade upgrading), and a comparable proportion were reassigned to poorly cohesive carcinomas (both signet-ring cell and non-signet-ring subtypes).

The subtype initially established by pathologists as tubular adenocarcinoma, high-grade, underwent changes in only 31% of cases: 21% were reclassified as poorly cohesive carcinoma, NOS, 6.9% were reassigned to tubular adenocarcinoma, low-grade, and 3.1% were classified as adenocarcinomas with mixed subtypes.

Another major histological type—poorly cohesive carcinoma, signet-ring cell subtype—was confirmed in 72.4% of cases and remained unchanged after reannotation. A significant proportion (17.3%) were reassigned to the poorly cohesive, non-signet-ring subtype. A small number of cases were reclassified as tubular adenocarcinoma, high-grade, mucinous adenocarcinoma, or adenocarcinoma with mixed subtypes. Other histological categories occurred either rarely or did not change after reclassification.

Poorly cohesive carcinomas are characterized by pronounced heterogeneity, and multiple studies suggest that grouping them into a single “diffuse carcinoma” category reduces diagnostic precision. Our annotations revealed that, of 185 carcinomas that should have been categorized as poorly cohesive, 33.5% had been initially misclassified as tubular adenocarcinomas (both low- and high-grade) or as adenocarcinomas with mixed subtypes.

In 2019, under the leadership of Fatima Carneiro, a European consensus on the subclassification of poorly cohesive gastric carcinomas was established [16]. According to this consensus, if ≥90% of the tumor consists of signet-ring cells, it should be classified as a poorly cohesive carcinoma, signet-ring cell subtype. If ≥90% consists of non-signet-ring poorly cohesive cells (e.g., histiocytoid and others), it should be classified as a poorly cohesive carcinoma, NOS. In cases with a mixed composition of both subtypes, the tumor should be referred to as a poorly cohesive carcinoma, combined subtype.

**Figure 3:**
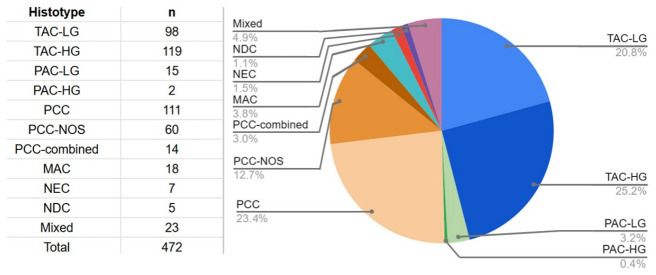
Distribution of histological types after annotation according to the WHO classification, with the inclusion of the subtype “poorly cohesive carcinoma, combined subtype” as proposed by F. Carneiro. Abbreviations: TAC-LG, tubular adenocarcinoma, low-grade; TAC-HG, tubular adenocarcinoma, high-grade; PAC-LG, papillary adenocarcinoma, low-grade; PAC-HG, papillary adenocarcinoma, high-grade; PCC, poorly cohesive carcinoma, signet-ring cell subtype; PCC-NOS, poorly cohesive carcinoma, NOS; PCC-combined, poorly cohesive carcinoma, combined subtype; MAC, mucinous adenocarcinoma; NEC, neuroendocrine carcinoma; NDC, undifferentiated carcinomas; Mixed, adenocarcinoma of mixed subtypes.

Based on these criteria, among the 185 poorly cohesive carcinomas identified in our dataset, the majority were signet-ring cell carcinomas (111 cases, 60%), followed by 60 non-signet-ring carcinomas (32.4%) and 14 combined subtypes (7.6%). Figure 3 illustrates the distribution of the most accurate histological tumor types of the stomach obtained after annotation and application of the WHO criteria.

According to Lauren’s classification, cases of diffuse-type carcinoma identified during the initial examination remained classified as such after reclassification based on annotation results in 98.2% of observations. Only in two cases was diffuse carcinoma reassigned, one to the indeterminate category (tubular adenocarcinoma, high-grade) and the other to adenocarcinoma of mixed subtypes. Carcinomas of the mixed subtype by Lauren’s classification, initially established in five cases, remained unchanged (100%); however, following annotation, an additional 17 cases were reassigned to this category, predominantly from indeterminate carcinomas. Intestinal-type carcinomas, initially diagnosed, retained their classification in 49.7% of cases after annotation, whereas 30.5% were reassigned to the indeterminate group, 16.7% to diffuse carcinomas, and 2.9% to mixed types. Among indeterminate carcinomas, 57.1% remained unchanged, while 27.9% were reclassified as diffuse type, 7.8% as intestinal type, and 7.1% as mixed type. Analysis of grade modifications revealed that, under the two-tier system, annotation altered the grade in 26.6% of cases, whereas under the three-tier system, changes were observed in 34.3% of cases.

In pursuit of refining classification approaches for established histological types, an attempt was made to assign the histological type based on the predominant histological component. Specifically, in the annotation dataset (where the area of each histological component was indicated), the component with the highest percentage was selected, and the case was classified accordingly. This approach, however, inherently excludes mixed tumors. Under this method, TAC-G1 and TAC-G2 were combined into TAC-LG, PAC-G1 and PAC-G2 into PAC-LG, PCC and PCC-NOS into PCC, and MAC1 with MAC2 into MAC. When these conditions were applied, tubular adenocarcinoma remained the most frequent histotype, accounting for 47.6% (as opposed to 46% under WHO criteria). The proportion of low-grade tubular adenocarcinomas increased from 20.8% to 29.4%, while the high-grade proportion decreased from 25.2% to 18.2%. This shift likely reflects the frequent co-occurrence of low- and high-grade components, resulting in grade adjustment in some cases rather than a change in histotype.

The second most frequent histotype remained poorly cohesive carcinoma. After combining PCC and PCC-NOS, their cumulative frequency amounted to 40.5%. Other histotypes remained nearly unchanged: papillary adenocarcinoma (4.4% vs. 3.2%), mucinous adenocarcinoma (4.4% vs. 3.8%), and neuroendocrine carcinoma (1.7%).

Overall, 61.4% of gastric adenocarcinomas comprised a single histological component regardless of classification approach, suggesting that attempts at improving classification pertain to slightly more than one-third of cases (38.6%). Within this subset, most adenocarcinomas consisted of two components (27.5%), while 11.0% combined more than two components (see Supplementary Materials Figure S9). It should also be emphasized that histological types were distributed primarily based on conventional histological evaluation, whereas carcinomas with neuroendocrine differentiation remain underrepresented. According to several studies, neuroendocrine differentiation occurs in more than 60% of cases, yet its detection requires comprehensive immunohistochemical analysis with markers of neuroendocrine lineage.

From an oncological standpoint, the most critical parameter is the assessment of patient survival outcomes. Detailed and accurate annotation enabled correlation of survival data with precise histological parameters. Survival information was obtained from the Federal Death Registry (RFS) of the Unified Medical Information System (EMIAS). Survival data were not available for all patients included in the study; therefore, below we present a comprehensive clinico-morphological characterization of tumors in patients for whom survival information was available (Table 1).

Univariate survival analysis demonstrated that patient age greater than 65 years was associated with an unfavorable prognosis in gastric carcinoma (Log Rank = 6309; *p* = 0.012). The cumulative one-year survival rate was 75% for patients younger than 65 years and 53% for those older than 65 years, respectively. The mean survival time for patients younger than 65 years was 20.2 months, which was significantly higher (*p* = 0.012) compared with 14.0 months for patients older than 65 years.

Due to the unequal distribution of patients across disease stages, further statistical analysis was performed after consolidating patients into three groups according to the extent of disease, following the SEER (Surveillance, Epidemiology, and End Result) registry classification [17]. Cases were categorized as localized, regional, or disseminated. Univariate survival analysis revealed a strong association between disease extent and prognosis (Log Rank = 30,768; *p* < 0.0001).

**Figure 4:**
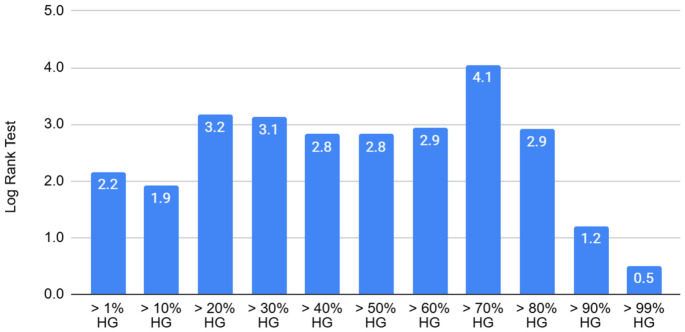
Comparison of thresholds for determining tumor grade depending on the proportion of the high-grade component (HG), based on Kaplan–Meier survival analysis.

Given the ambiguity in assessing the degree of differentiation in gastric carcinoma, and in order to identify a threshold value for stratifying tumors into high-grade and low-grade categories based on prognostic significance, all carcinomas were “decomposed” into tumors with a high-grade component ranging from 1% to 99% (Figure 4).

Statistically significant results were observed when the high-grade component of the tumor exceeded 70%. Survival in patients with such tumors was significantly higher (*p* = 0.044) compared with all other thresholds. Notably, tumors with low thresholds of 1% and 15% demonstrated markedly poorer outcomes. This suggests that tumors with a predominant high-grade component (greater than 70%) may exhibit increased aggressiveness. The 70% threshold is also practical for clinical use and may even be applied without precise annotation (covering approximately two-thirds of the tumor area). However, this result was obtained in an exploratory study, rather than a confirmatory one. Future studies will aim to validate the 70% threshold in an independent cohort.

Precise annotation allows for visualization not only of the diversity of tumor histotypes in gastric carcinoma but also of the proportion of pre-neoplastic changes and non-invasive carcinomas accompanying invasive growth. Without precise annotation, these features would at best be noted only in micro-descriptions, yet, as demonstrated in this study, they are frequently overlooked when invasive carcinomas are detected.

High-grade dysplasia within the same biopsy as an invasive carcinoma was observed in 39 cases (8.2% of all carcinomas) and co-occurred with the following carcinoma types: tubular adenocarcinoma, low-grade—22 cases (46%); tubular adenocarcinoma, high-grade—9 cases (19%); poorly cohesive carcinoma, signet-ring cell subtype—4 cases (8%); adenocarcinoma with mixed subtypes—2 cases (4%); and one case each of mucinous adenocarcinoma and low-grade papillary adenocarcinoma. The proportion of high-grade dysplasia ranged from 1% to 86% of the tumor area. Specifically, 20 cases had 1–9%, 12 cases had 10–49%, and 7 cases had more than 50% high-grade dysplasia.

Low-grade dysplasia alone was detected in 48 cases and co-occurred with the following carcinoma types: tubular adenocarcinoma, low-grade—18 cases (38%); tubular adenocarcinoma, high-grade—16 cases (33%); poorly cohesive carcinoma, signet-ring cell subtype—8 cases (17%); and two cases each of poorly cohesive carcinoma, non-signet-ring subtype, low-grade papillary adenocarcinoma, and adenocarcinoma with mixed subtypes. The proportion of low-grade dysplasia ranged from 1% to 99%, with 23 cases having 1–9%, 19 cases 10–49%, and 6 cases over 50%.

Overall, dysplasia (low- and/or high-grade) was observed in 74 cases (15.7%), with proportions ranging from 1% to 99% (mean 25%). Specifically, 34 cases had 1–9%, 25 cases 10–49%, and 15 cases over 50%.

In histological types such as undifferentiated carcinoma, high-grade papillary adenocarcinoma, and neuroendocrine carcinoma, dysplastic changes were not observed. This finding may have fundamental implications for understanding gastric cancer pathogenesis, suggesting that these forms may arise de novo or that dysplastic changes were not captured in the biopsy. Further investigation is required, and digital pathology offers the tools to address this question.

In addition to dysplastic changes, metaplastic changes warrant attention. Any proportion of intestinal metaplasia (>1%) observed in the scanned slide was recorded. Overall, intestinal metaplasia was detected in 89 cases (18.9%).

Intestinal metaplasia was more frequently observed in males than in females (26.6% vs. 10.7%; *p* = 0.0002). Additionally, intestinal metaplasia was more commonly detected in intestinal-type adenocarcinomas according to Lauren’s classification (23.8% in intestinal-type adenocarcinomas versus 11.3% in diffuse-type carcinomas). Intestinal metaplasia was significantly associated with the presence of low- or high-grade dysplastic components within the tumor, being observed in 36% of carcinomas with dysplasia, compared with 11% in those without dysplasia (*p* < 0.001).

Another important parameter, frequently overlooked by pathologists in routine practice and typically reported only in microscopic descriptions, is ulceration. Through precise annotation of fibrin (F) and granulation tissue (GT) classes in gastric biopsy specimens, it became possible to assess the proportion of tumors exhibiting ulceration. Overall, ulceration was detected in 28.2% of gastric carcinomas.

Based on the study findings, criteria for a multifactorial prognostic index were established, incorporating patient age and significant morphologic parameters readily available for detailed evaluation in gastric biopsies. These criteria include:

- Age > 65 years (1 point);
- High-grade component exceeding 70% of tumor area (1 point);
- Tumor ulceration: presence of >1% fibrin or granulation tissue (1 point);
- Absence of intestinal metaplasia in adjacent mucosa (1 point);
- Absence of dysplasia in adjacent mucosa (1 point).

Each parameter was assigned 1 point, enabling stratification of patients into high-risk, intermediate-risk, and low-risk groups. This three-tier system was also converted into a two-tier system for practical application. Both systems are illustrated in Table 2.

Univariate survival analysis confirmed that patients in the high-risk group (4–5 points) exhibited a significantly poorer prognosis compared with those in the low-risk group (0–3 points) among patients with gastric carcinoma (Log Rank = 14,754; *p* < 0.0001). One-year cumulative survival was 71% in the low-risk group and 40% in the high-risk group, respectively. The mean survival time for patients in the low-risk group was 19.2 months, which was significantly longer than the 11.3 months observed in the high-risk group.

**Figure 5:**
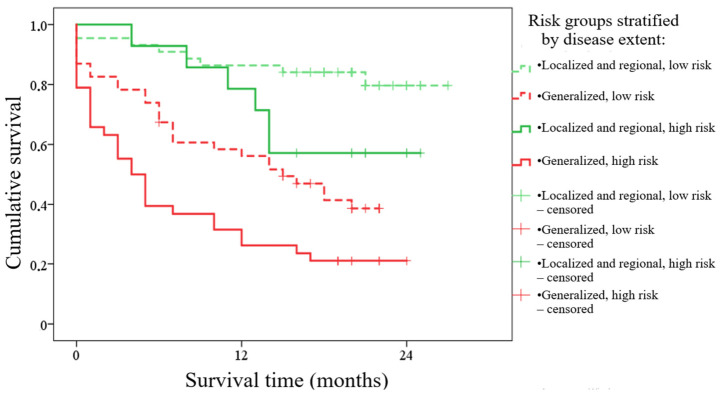
Survival curves for low- and high-risk groups stratified by disease stage: stage I–III (localized and regional) vs. stage IV (generalized).

Thus, the proposed point-based prognostic index, derived from a combination of the most prognostically significant factors, demonstrates several advantages over conventional histological classifications (Figure 5).

Univariate survival analysis stratified by disease extent further demonstrated that within each stage, survival in the high-risk group remained inferior to that in the low-risk group (Log Rank = 39,500; *p* < 0.0001). Among patients with localized or regional gastric cancer (corresponding to Stages I, II, and III), mean survival was 23.4 months in the low-risk group, significantly higher (*p* < 0.0001) than the 18.8 months observed in the high-risk group. Similarly, among patients with generalized (metastatic) gastric cancer corresponding to Stage IV, mean survival was 13.1 months in the low-risk group, significantly exceeding the 8.4 months observed in the high-risk group (*p* < 0.0001).

In total, 80% percent of the annotated slices were used to train the neural network (NN), while the remaining 20% were reserved for control tests with pre-defined classes. The performance of the trained NN was evaluated using a confusion matrix. Predicted values were classified as positive or negative, whereas actual outcomes were categorized as true or false [18]. This approach enabled an assessment of predictive accuracy both qualitatively and quantitatively, thereby allowing measurement of prediction error. Among non-neoplastic classes, the highest prediction accuracy was achieved for fibrin (0.86), followed by normal glands and intestinal metaplasia. Among carcinoma classes, the highest prediction accuracies were obtained for tubular adenocarcinoma G3 (0.91), poorly cohesive carcinoma, papillary adenocarcinoma G1 (0.88), signet-ring cell carcinoma (0.87), and tubular adenocarcinoma G1 (0.84). Mucinous adenocarcinoma, poorly cohesive carcinoma NOS, and tubular adenocarcinoma G2 achieved moderate prediction probabilities of approximately 0.7 (0.66–0.71). Other tumor classes yielded lower predictive values, reflecting their lower frequency. Similarly, dysplastic changes showed low predictive values, with high-grade dysplasia at 0.53 and low-grade dysplasia below 0.1. Nevertheless, these results indicate that for several classes, the prediction probability is sufficiently high to enable evaluation of the developed AI system on a test dataset.

The test set of gastric biopsies consisted of randomly selected scans from 250 patients who had undergone gastric biopsy. A total of 775 fragments were analyzed, with an average of 3 fragments per scan (mean = 3.1; range 1–5 fragments per scan). The clinicopathological characteristics of patients in the test set were comparable to those in the training set.

Expert review of the scanned histological slides led to reclassification in several cases: eight instances of high-grade dysplasia were reclassified as invasive carcinoma, and two instances of low-grade dysplasia were also upgraded, resulting in a total of 108 carcinomas instead of the original 100. Among invasive carcinomas, 62.8% were identified as tubular adenocarcinomas (33.3% low-grade, 29.5% high-grade), 18.1% as poorly cohesive carcinomas (14.3% signet-ring cell subtype, 3.8% non-signet-ring cell subtype), 14.3% as papillary adenocarcinomas (11.4% low-grade, 2.9% high-grade), 4.8% as mucinous adenocarcinomas, and 3.8% as adenocarcinomas with mixed subtypes.

The distribution of histological types in the test set, as determined by experts, correlated closely with the distribution in the training set, which had been classified according to the WHO histological classification following annotation. The proportion of tubular adenocarcinomas was nearly identical across both datasets, while the proportion of poorly cohesive carcinomas in the test set was slightly lower, and the proportion of papillary adenocarcinomas was slightly higher. These distributions are consistent with WHO data, indicating that with proper tumor classification, reproducibility can be achieved, ultimately allowing for more precise stratification of patients into prognostic groups.

Analysis of the “errors” made by pathologists revealed that, out of 250 cases, there were 5 cases where three specialists (the auditor and two experts) disagreed with the initial opinion; such errors might be regarded as more serious. In 7 cases, the auditor’s opinion disagreed with one of the experts, representing less “serious” discrepancies associated with the subjectivity of opinions, though this does not diminish the need for a more thorough analysis. Finally, in 11 cases, the final opinion of the expert group diverged from the initial opinion, accounting for 4.4% of all studies included in the analysis (Table S37 in the Supplementary Materials).

To evaluate how diagnostic discrepancies might have impacted patient outcomes and to ensure that an initially inaccurate diagnosis did not compromise medical care, a comprehensive review was conducted in collaboration with the attending physicians, including monitoring of patient follow-up. Among 11 patients with discrepancies, 2 underwent repeat biopsies, which confirmed the diagnoses established by the expert panel. Submucosal resections were performed in 7 patients, with the surgical findings concordant with the expert panel in 5 cases: in three instances, high-grade dysplasia was reclassified as early carcinoma, and in two, high-grade dysplasia in the resected specimen was interpreted as low-grade dysplasia. The remaining two cases were classified as overdiagnoses; however, as patient monitoring was indicated regardless, case review was limited to discussions with the attending physicians. In two cases where the expert panel’s assessment differed from the submucosal resection findings but aligned with the initial diagnosis, it was concluded that the high-grade dysplasia regions identified in the biopsy may have been entirely excised in the biopsy specimen, leaving only the low-grade adenoma component in the resected tissue. Revisions were documented in the study protocols, and the information was communicated to the treating physicians and patients to enhance vigilance and ensure close follow-up. Additionally, two patients initially diagnosed with high-grade dysplasia underwent gastric resection due to clinical suspicion of carcinoma, and in both cases, tubular adenocarcinoma, low-grade pT1a, was identified.

Thus, the review of cases revealed that discrepancies between initial biopsy interpretations and expert panel evaluations did not result in deficiencies in medical care; however, more precise diagnostics could have shortened patient evaluation times and, in some cases, prevented additional interventions.

Each scan from the test set was uploaded to the software developed in collaboration with Innopolis University. In addition to tabular data, a PNG file with a 1000 × 1000-pixel thumbnail was generated for each scan, enabling visualization of the specific areas where the AI algorithm detected the annotated classes.

The determination of histological types and ICD-10-CM codes based on manual annotations (precise morphometry/mapping of all histological patterns in a scan) versus AI algorithm output requires fundamentally different approaches. The AI model, when applied to new images, demonstrates reduced accuracy in classifying all categories. Moreover, implementation of multiple neural networks is required even for seemingly simple tasks. This underscores that approaches used to assign ICD-10-CM codes—where any proportion of invasive carcinoma is classified as C16.9—cannot be directly applied to AI algorithms.

Analysis of histological structures demonstrated that the proportion of tumor tissue relative to non-neoplastic tissue for C16.9 and D00.2 is comparable: 29% for C16.9, 21% for D00.2, 15% for D13.1, and 16% for K29.7. Similarities were also noted between D13.1 and K29.7. Consequently, it is not yet possible to reliably separate the four diagnoses within the available datasets. Therefore, invasive and in situ carcinomas were combined into a single gastric carcinoma group (C16.9 + D00.2). Clinically, this consolidation is reasonable, as even among pathologists, distinguishing invasive from non-invasive carcinoma can be challenging, which is reflected in the lowest sensitivity and specificity observed during initial expert diagnosis assessments.

Inter-expert agreement analysis comparing initial interpretations with expert panel assessments showed that for C16.9 versus non-C16.9, sensitivity was 92.6% and specificity 100% (Cohen’s Kappa = 0.934, *p* < 0.001). When comparing C16.9 + D00.2 versus D13.1 + K29.7, inter-expert agreement improved, with sensitivity of 99.2% and specificity of 98.4% (Cohen’s Kappa = 0.976, *p* < 0.001).

Using the same principle, a benign gastric mucosal change group was delineated (D13.1 + K29.7). Following the consolidation of these groups, a new algorithm was developed for the classification of gastric carcinomas (both invasive and non-invasive) based on the detected histological classes.

The analysis of inter-expert concordance between the expert group and the AI algorithm in classifying biopsies as invasive carcinomas versus all other diagnoses demonstrated a sensitivity of 63.8% and a specificity of 78.2%. When the classification was expanded to carcinomas (both invasive and non-invasive) versus benign pathology (adenomas and inflammatory changes), sensitivity increased to 78.3% with a specificity of 75.3%.

The main improvement in algorithm performance was achieved through the creation of a three-level ensemble of neural networks, as described in the Section 2. This process enables the algorithm to assign each detected pattern to its corresponding group and thereby establish the final ICD code. The trained classes were grouped according to the nature of pathology changes, and a color-coding scheme was implemented to facilitate visualization. Non-neoplastic classes were assigned shades of green; low- and high-grade dysplasia, as well as features of ulceration (fibrin and granulation tissue), were assigned shades of blue; and carcinoma classes were subdivided according to Lauren’s classification, depicted in shades of red in the AI-generated analysis images.

**Figure 6:**
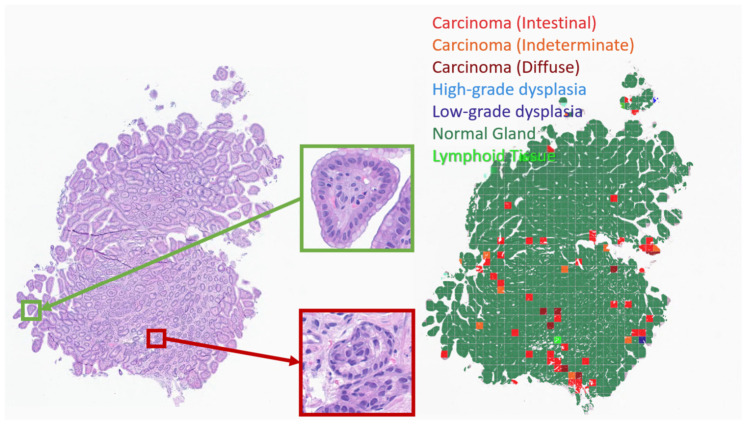
Example of the trained AI algorithm applied to a gastric biopsy (K29.7). Left—original scan, magnification ×50 (highlighted areas at ×400); right—result of the algorithm, magnification ×50.

Figure 6 illustrates an example of AI-based analysis of a gastric mucosal biopsy with inflammatory changes. The algorithm labeled the majority of the scan area in green, corresponding to non-neoplastic tissue. Nevertheless, certain slices were classified by the AI as dysplasia or invasive carcinoma. Upon closer examination, some of these slices (red area) corresponded to altered glands with regenerative atypia in the setting of inflammation, which the AI interpreted as carcinoma. It should be noted that, out of context, such 256 × 256 pixel slices may also appear as adenocarcinoma to the pathologist’s eye.

**Figure 7:**
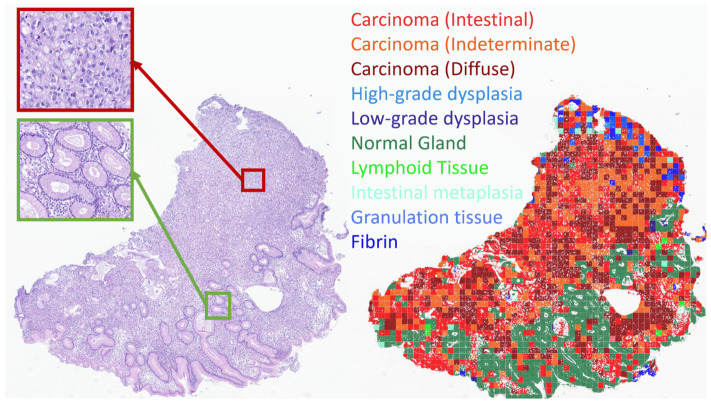
Example of the trained AI algorithm applied to a gastric biopsy with carcinoma (C16.9). Left—original scan, magnification ×50 (highlighted areas at ×400); right—result of the algorithm, magnification ×50.

A demonstrative example of a biopsy containing carcinoma is illustrated in Figure 7. Unlike the previous image, in this scan, the majority of the area is represented by invasive carcinoma classes. Normal glands and non-neoplastic changes were also reliably recognized by the algorithm. However, further development is required to improve the precise determination of histological subtypes. Although the diffuse-type carcinoma was identified across a significant area by the algorithm, approximately the same slices were simultaneously classified as intestinal-type carcinoma or as indeterminate, highlighting the need for refinement in subtype discrimination.

Based on the obtained data, an inter-expert agreement analysis was conducted. Comparisons were made between the primary pathologist’s assessment and the AI, between the auditor’s assessment and the AI, and between the expert group’s evaluation and the AI, with the expert group’s diagnosis considered decisive.

Currently, the sensitivity of inter-expert agreement between the primary assessment and the AI is 73.3% when divided into cancer vs. non-cancer, and 81.5% when divided into malignant vs. benign processes; specificity is 82.6% and 81%, respectively (Table 3).

**Figure 8:**
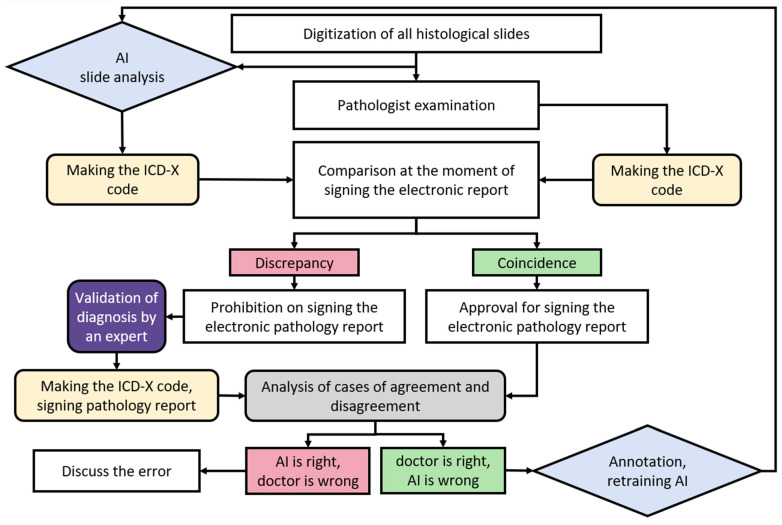
Algorithm for the application of AI systems to prevent medical errors.

AI systems can assign ICD-10 codes in parallel with pathologists, and in cases of discrepancy, the digitized histological specimen can be submitted for diagnostic validation by a more experienced pathologist. A detailed algorithm is presented in Figure 8.

## 4. Discussion

A review of the literature revealed that many researchers have sought to classify gastric cancer samples using pathology slides, and numerous studies have reported impressive results. In discussing our findings, we will identify the aspects in which our model was more successful and those in which it may still require refinement.

In our study, the system was implemented as a three-level ensemble: Level 1: Cancer/Other pathology/Normal; Level 2: Morphological type; Level 3: Assignment of ICD-10 code. This architecture achieved a sensitivity of 79.6% and a specificity of 86.7% in differentiating malignant from benign lesions in an independent test cohort. Other researchers have also used staged (“cascade”) approaches, confirming the effectiveness of this strategy. Cho et al. employed a two-stage process, with a design that included CNN-1 (tumor/normal) and CNN-2 (histological subtype: adenocarcinoma or not). The similarity lies in the initial filtering of normal tissue, followed by detailed subtyping. This resulted in exceptional accuracy—an AUROC of 1.000 during internal validation. This demonstrates that decomposing the task into stages minimizes errors [19]. Zhu et al. also applied a two-stage system, combining DCNN and Graph Convolutional Networks (GCN) for classification according to WHO standards. This is practically equivalent to our transition to ICD-10 codes, as ICD is based on the WHO classification. The result was an AUC of 0.979 [20]. Hu et al. used a cascade system (LN-LNM) for detecting lymph node metastases, employing a cascade detection and quantification system. The result was high quantification accuracy (97.13%) and reliability in identifying micrometastases [21].

The GastroMIL system (Huang et al.) also used a two-stage scheme (tile analysis followed by aggregation via RNN). This approach achieved an accuracy of 0.920 in diagnosing gastric cancer on external validation, comparable to expert pathologists [22]. It should be noted that the transition to a multi-level architecture is often driven by the need to surpass the performance of single models. In our research, we observed that attempts to train a single neural network for direct assignment of ICD-10 codes encounter difficulties, as cancer and dysplasia often have similar proportions of tumor tissue. The use of an ensemble specifically allowed for structuring these data. The study by Cho et al. explicitly states that their two-stage process yielded better results than attempts to train a multiclass model in a single step [19].

Thus, a similar structure (cascade or multi-level) was used by Cho et al., Zhu et al., and Huang et al. [19,20,22]. In all cases, this design enabled the achievement of high results (accuracy/AUC > 0.90), as it mimics physician logic: “identify a problem → determine the type → establish a diagnosis.”

The distinction and advantage of our study is the addition of a third level (ICD-10), making the system more complete for clinical application, whereas most other works stop at the morphological type. It is also important to highlight our meticulously annotated dataset of 970 slides, with annotations covering 90% of the area across more than 20 classes. This represents a uniquely detailed data array that significantly surpasses many existing analogs in depth of processing. Our approach to annotating over 20 histological classes—including tumor subtypes, dysplasia grades, and metaplasia—is extremely detailed compared with most studies, which focus on simplified classification. For example, the study by Song et al. used a larger dataset (2123 slides), but annotation was performed at the pixel level using a simplified scheme: only “malignant,” “benign,” or “ignore.” Unlike our work, they did not distinguish more than 20 specific morphological subtypes within these groups [23].

In the study by Ma et al., a dataset of 763 slides was used, with annotation limited to three broad categories: normal mucosa, chronic gastritis, and intestinal-type adenocarcinoma. In contrast, our design allows the model to identify much finer distinctions, such as signet-ring cell carcinoma, neuroendocrine structures, or various grades of dysplasia [24].

In the study by Fu et al., the StoHisNet architecture was trained to classify only four subtypes (tubular, mucinous, papillary adenocarcinoma, and non-tumor tissue), which also falls significantly short of our classification in terms of the breadth of morphological forms covered [25].

In 2023, an interesting study with a similar design was published, in which histological parameters were correlated with disease prognosis; however, these parameters were obtained not through manual annotation but through the application of an AI algorithm. It is likely that manual annotation is too labor-intensive for routine clinical practice, whereas AI-driven analysis may substantially enhance the efficiency and accuracy of pathologists in real-world settings [26].

Undoubtedly, annotating 90% of the entire biopsy area is a labor-intensive approach that differs significantly from the widely used Weakly Supervised Learning method. In the study by Huang et al. (GastroMIL), a fundamentally different design was employed: the authors used Multiple Instance Learning (MIL) to deliberately avoid labor-intensive pixel-by-pixel annotation. They trained the model using only overall slide diagnoses (labels of bags), arguing that manual annotation is too cumbersome and hinders AI implementation. Our results, by contrast, demonstrate the value of Strong Supervision, as detailed annotation enabled the first quantitative assessment showing that up to 35.3% of the area in cancer biopsies is actually occupied by non-neoplastic tissue [22]. The study by Liao et al. (HER2Net) also used a pixel-level tumor detector, but its aim was only the segmentation of tumor regions for subsequent HER2 status assessment, not the detailed typing of all tissue [27].

Thanks to this annotation design, we obtained results with direct significance for molecular diagnostics. Literature indicates that a tumor-to-normal tissue ratio exceeding 20% is required for reliable genetic testing [28]. Our detailed annotation enables the AI to automatically select fragments with the highest tumor cell content for analysis, a feature not implemented in studies with simpler annotation, such as the work by Park et al., where classification was based on three broad categories without precise calculation of component proportions [29].

Thus, our dataset stands out in global research due to its unprecedented annotation density (90% of area) and morphological depth (over 20 classes). While other groups (Huang et al. [22]) aim to minimize pathologists’ workload through weak supervision, our design relies on high-precision expertise, enabling the solution of more complex tasks, such as automatic assignment of ICD-10 codes and precise risk stratification.

The results of our study regarding the quantitative composition of tumors and the revision of histological classification open new perspectives for understanding gastric cancer heterogeneity. The use of detailed annotation (covering 90% of the slide area) revealed data that previously escaped attention during standard pathology examination.

The finding that 33.5% of poorly cohesive carcinomas (PCC) were erroneously classified as tubular adenocarcinoma (TAC) highlights the problem of subjectivity in the “gold standard.” A similar challenge was noted in the study by Cho et al.: poorly cohesive carcinomas in H&E-stained images appear as scattered bright spots, mimicking inflammatory infiltrate. This explains why pathologists may mistakenly interpret them as other tumor types or even as non-specific inflammation upon cursory examination [19]. The study by Veldhuizen et al. demonstrates an even higher level of discordance: in their work, the AI classifier identified 54% of samples as intestinal type, while pathologists initially classified them as diffuse type (65%) [26]. This supports our conclusion that detailed annotation according to WHO criteria significantly reduces subjectivity and refines histotype classification. Furthermore, we showed that low-grade tubular adenocarcinomas are often reclassified as high-grade (TAC-HG) after annotation (30.8% of cases in our study), as AI more accurately assesses the proportion of the high-grade component. In addition, detailed annotation in our study enabled the system to detect “hidden” components, such as microfoci of dysplasia or metaplasia within carcinomas, which accompany tumor growth in 61.6% of cases but are often not recorded in the final ICD code.

During our research, a prognostically significant threshold for the high-grade component was identified: a threshold of >70% high-grade component in the tumor was statistically significantly associated with worse survival compared with other thresholds (1%, 15%, etc.) (*p* = 0.044). According to the 2019 WHO classification, if a predominantly low-grade tumor contains even a focal high-grade component, the tumor is classified as mixed and assigned a high-grade. The threshold value of 70% should be interpreted as a preliminary finding, as it has not been validated in an independent cohort (which is planned for future research). The use of “areal” or “proportional” characteristics for prognosis is supported by other studies. For example, Wang et al. showed that a quantitative parameter—the ratio of tumor area to metastatic lymph node area (T/MLN)—is an independent prognostic factor. This supports a general scientific trend: prognosis depends not only on the presence of a feature but also on its quantitative extent in the tissue [30]. In the study by Chen et al., when developing the PSGC signature, quantitative parameters of feature distribution in H&E-stained slides were also considered, enabling more accurate risk stratification than the standard TNM stage [31].

The data obtained also have high clinical significance, as they directly correlate with the survival data collected during the study. In our research, patients in the high-risk group (which includes the criterion of >70% high-grade component) had a mean survival of 11.3 months, while the low-risk group had a mean survival of 19.2 months. Such clear stratification (*p* < 0.0001 for the entire scoring system) demonstrates that the 70% threshold is a reliable biological marker of tumor aggressiveness. While other systems, such as the DLPS signature by Zhang et al. [32] or the PSGC signature by Chen et al. [31], use complex mathematical models for prognosis, our approach provides an understandable and reproducible morphological criterion enabled by detailed pixel-by-pixel annotation of 90% of the biopsy area. This makes the model explainable and more suitable for implementation in clinical practice.

It should be noted that in our study, even among patients with metastatic cancer (stage IV), the proposed model effectively identified a subgroup with an extremely unfavorable prognosis (median survival of only 8.4 months versus 13.1 months in the low-risk group). This trend is confirmed by Wei et al.: integrating pathology features with clinical data increased the survival C-index from 0.660 to 0.744, highlighting the need to go beyond the TNM system alone [33]. Our developed multi-level ensemble of convolutional neural networks demonstrated moderate but statistically significant diagnostic accuracy on an independent test set. When differentiating malignant (carcinoma in situ and invasive) from benign processes, the algorithm achieved a sensitivity of 79.6% and a specificity of 86.7% (Cohen’s Kappa = 0.656, *p* < 0.001). The agreement between the algorithm and expert consensus (Kappa = 0.581) was lower than inter-pathologist agreement (Kappa = 0.934–0.976), indicating the current auxiliary, rather than replacement, status of the system.

Our sensitivity and specificity indicators fall within the range reported for systems intended for clinical use, where balancing the detection of pathology and minimizing false positives is important. In the study by Song et al., a system with extremely high sensitivity (close to 100%) but lower average specificity (80.6%) was developed using a real-world dataset. Our results show slightly lower sensitivity but noticeably higher specificity (86.7%), which is crucial for reducing pathologists’ workload in reviewing false positives [23]. In the study by Wang et al. [30], a two-stage model achieved an overall accuracy of 86.5% on a test set of 200 slides. This is comparable to your data and confirms that when transitioning from individual “tiles” (fragments) to whole slide image (WSI) analysis, accuracy typically stabilizes at the 80–90% level. In contrast, studies using “clean” public datasets (e.g., GasHisSDB), such as those by Mudavadkar et al. [34] and Zubair et al. [35], report accuracies of 97–99%. However, such high figures are often associated with pre-selected patches rather than complex biopsies with artifacts, highlighting the value of your validation on an independent test set.

At the same time, our result for agreement (Kappa 0.581 vs. 0.934–0.976 for pathologists) is a key argument in favor of the auxiliary status of AI. The study by Cho et al. also found that the standalone accuracy of pathologists without AI was significantly lower than that of the model itself, but the collaborative approach (pathologist + AI) yielded the best result, increasing pathologists’ accuracy from 0.63–0.78 to 0.76–0.97. This supports your conclusion that the system should not replace the pathologist but serve as a decision support tool [19]. The study by Park et al. notes that using AI reduced slide review time by 47% while providing 100% sensitivity for epithelial tumors. This aligns with your position: AI is effective as a “sieve” for preliminary screening, allowing the pathologist to focus on complex cases [29].

According to the literature, AI systems often make errors in cases with pronounced inflammation, necrosis, or preparation artifacts (tissue folds), which pathologists easily interpret. This explains why inter-pathologist agreement (Kappa > 0.9) remains the “gold standard,” unattainable for algorithms alone [23,36]. Thus, our results confirm the general scientific trend: modern multi-level CNN ensembles have reached a point where they can reliably safeguard pathologists from missing malignant processes (demonstrating high specificity and significant accuracy), but their current role remains internal quality control and providing a “second opinion.” The high inter-expert agreement in your test (Kappa up to 0.976) further emphasizes the difficulty for AI in achieving the level of human intuition in morphology.

Finally, analysis of the algorithm’s performance revealed its strengths (reliable recognition of normal and non-neoplastic tissue) and critical limitations (overdiagnosis of cancer on artifacts, low accuracy for rare classes and tumor subtypes, and difficulties in distinguishing dysplasias). This is consistent with data from Song et al., whose system showed nearly 100% sensitivity, effectively “filtering out” normal tissue so that the pathologist could focus on suspicious areas. Furthermore, in that study, folds, knife marks, and restaining were also identified as the main causes of false positives [23]. The low accuracy for dysplasias (<0.1 for low-grade) observed in our work is a common challenge: even for expert pathologists, distinguishing regenerative atypia, metaplasia, and dysplasia is the most frequent cause of diagnostic discrepancies [36].

The identified discrepancy rate of 4.4% (11 cases out of 250) in routine practice is a significant indicator. The study by Wang et al. found that 4.6% of cases were initially under-staged by pathologists due to missed micrometastases detected by AI [30]. Thus, our 4.4% confirms that the AI system is capable of detecting errors made by humans due to fatigue or complex morphological patterns. Therefore, discrepancies do not always result in fatal errors, but their elimination reduces patient examination time and prevents unnecessary interventions.

At the same time, the concept of parallel ICD-10 coding and automatic validation of discrepancies by an expert aligns with the “second opinion” approach described in the literature, indicating that the highest accuracy (up to 91.2%) can be achieved. The model we propose for the automatic referral of contentious slides to a senior expert transforms AI from a “black box” into an objective tool for internal audit. This addresses the ethical issue of accountability by preserving the pathologist’s ultimate decision-making authority while using AI as a “safety net” system.

### Limitation

- The median overall survival could not be determined for the study groups due to the limited follow-up period. Therefore, mean survival time and the percentage of 1-year survival were used as reference points instead.
- Certain annotation classes (fibrotic/desmoplastic stroma and reactive/regenerative epithelial changes) were not included in the initial dataset. This omission negatively affected the model’s accuracy, particularly in cases with histotype PCC-NOS. Future work will focus on improving the model by annotating these additional pathology classes.
- Survival data were not available for all patients (only for 142 patients), as this was not an obligatory inclusion criterion for the study. Additionally, due to the limited statistical power of the study, it was not possible to perform Cox regression analysis or to conduct subgroup analyses according to treatment type.
- The AI model has not undergone external validation. Although a test dataset was used, all data were obtained from a single institution. The study results are planned to be validated in a multicenter study.

## 5. Conclusions

This study demonstrates the potential synergy between digital pathology and artificial intelligence. The developed prognostic index, based on biopsy assessment, enables early identification of patients with an unfavorable prognosis for personalized management strategies. The artificial intelligence algorithm has proven to be a reliable auxiliary tool, enhancing pathologists’ efficiency and reducing diagnostic subjectivity through automated quality control. A key practical conclusion is the proposed integration model, in which artificial intelligence acts as a “digital auditor,” directing the expert’s attention to contentious cases and optimizing the clinical workflow without radical restructuring. Thus, this work contributes to shaping a new paradigm of intelligent pathological diagnostics, focused on improving the accuracy, reproducibility, and prognostic value of morphological examination.

## Figures and Tables

**Table 1:** Clinicopathological parameters and overall survival (OS) in patients with gastric cancer (*n* = 472).

Clinico-Pathological Parameter	Total Patients (*n* = 472)	Patients in OS Group (*n* = 142)	Mean Survival (Months, 95% CI)	1-Year Survival (%)	Log Rank	*p*-Value
Sex						
Male	240	71	14.8 (14.7–18.4)	58	0.008	0.928
Female	232	71	16.7 (14.2–19.2)	62		
Age						
≤65 years	163	44	20.2 (17.3–23.2)	75	6309	0.012
>65 years	309	98	14.0 (12.0–16.1)	53		
Disease extent						
Localized	108	33	24.6 (22.4–26.9)	91	30,768	<0.001
Regional	82	25	18.5 (15.2–21.9)	76		
Generalized	282	84	11.4 (9.3–13.5)	43		
Lauren histotype (before annotation)						
Intestinal	203	60	18.0 (15.6–20.4)	73	5907	0.052
Undetermined	154	32	13.6 (10.8–16.4)	50		
Diffuse	110	50	14.9 (10.8–19.0)	50		
Mixed	5	0				
Grade (before annotation)						
Low-Grade	192	59	18.0 (15.6–20.4)	74	5674	0.017
High-Grade	270	80	14.6 (12.1–17.0)	50		
Not specified	10	3				
Lauren histotype (after WHO annotation, including ≥1% High-Grade)						
Intestinal	113	36	17.0 (14.1–19.9)	75	1711	0.425
Undetermined	151	53	16.7 (13.8–19.5)	60		
Diffuse	185	47	13.8 (10.8–16.9)	49		
Mixed	23	6	14.0 (8.3–19.6)	52		
Grade (after WHO annotation, including ≥1% High-Grade)						
Low-Grade	113	39	17.3 (14.4–20.3)	74	0.751	0.386
High-Grade	359	103	15.8 (13.6–18.0)	54		
PD-L1 status						
Negative	88	29	18.8 (14.9–22.7)	68	0.491	0.484
Positive	67	21	15.9 (11.9–19.9)	60		
N/A	317	92	14.4 (12.3–16.4)	55		
HER2 status						
Negative	140	40	18.2 (15.0–21.4)	64	0.027	0.870
Positive	15	10	17.7 (12.2–23.2)	73		
N/A	317	92	14.2 (12.1–16.3)	54		
Association with dysplasia, based on annotation						
<1% dysplasia	398	103	15.0 (12.8–17.2)	52	5027	0.025
>1% dysplasia	74	39	19.3 (16.7–21.9)	79		
Association with intestinal metaplasia, based on annotation						
<1% metaplasia	383	89	13.7 (11.6–15.9)	54	7908	0.005
>1% metaplasia	89	53	19.9 (17.2–22.7)	70		
Association with ulceration (GT and F), based on annotation						
<1% GT and F	322	88	17.7 (15.3–20.0)	64	3898	0.041
>1% GT and F	150	54	13.8 (11.2–16.5)	54		
Association with normal mucosa (NG), based on annotation						
<1% NG	367	103	15.0 (12.8–17.2)	50	5027	0.025
>1% NG	105	39	19.3 (16.7–21.9)	73		
Association with lymphoid aggregates (LT), based on annotation						
<1% LT	405	111	15.2 (13.3–17.2)	59	0.062	0.804
>1% LT	67	31	17.6 (14.1–21.2)	65		

N/A = not applicable (analysis not performed).

**Table 2:** Developed prognostic systems for predicting mortality risk in patients with histologically verified gastric carcinoma based on biopsy analysis.

Three-Tier System	Point	Two-Tier System	Point
Low-risk group	0–2	Low-risk group	0–3
Intermediate-risk group	3	High-risk group	4–5
High-risk group	4–5		

**Table 3:** Evaluation of inter-rater agreement between the initial (primary) diagnosis, the diagnosis of the AI algorithm, and the diagnosis of the expert group in gastric biopsies.

Parameter	Analysis Between Primary Diagnosis and Expert Group Diagnosis		Analysis Between Primary Diagnosis and AI Diagnosis		Analysis Between Expert Group Diagnosis and AI Diagnosis	
	C16.9 vs. Non-C16.9	C16.9 + D00.2 vs. D13.1 + K29.7	C16.9 vs. Non-C16.9	C16.9 + D00.2 vs. D13.1 + K29.7	C16.9 vs. Non-C16.9	C16.9 + D00.2 vs. D13.1 + K29.7
Cohen’s Kappa	0.934	0.976	0.559	0.624	0.581	0.656
*p*	<0.0001	<0.0001	<0.0001	<0.0001	<0.0001	<0.0001
Sensitivity, %	92.6	99.2	73.3	81.5	78.2	79.6
Specificity, %	100	98.4	82.6	81.0	80.5	86.7

## Data Availability

The original contributions presented in this study are included in the article. Further inquiries can be directed to the corresponding author.

## Supplementary Materials

The following supporting information can be downloaded at: https://www.mdpi.com/article/10.3390/jcm15093370/s1.
