# Supplementary material for: Artificial Intelligence for Tumor Tissue Detection in Stomach Cancer: A Retrospective Algorithm Development and Validation Study
Source: J Clin Med. 2026 Apr 28;15(9):3370. doi: 10.3390/jcm15093370 (PMC13163764; doi:10.3390/jcm15093370)
Supplement: Supplementary file 1 [file jcm-15-03370-s001.zip › jcm-4169956-supplementary.pdf]

Table S1 – Histological structures for annotation gastric biopsies

NG	Normal glands
IM	Intestinal metaplasia
LT	Lymphoid tissue
F	Fibrin
GT	Granulation tissue
GINL	Low-grade glandular intraepithelial neoplasia
GINH	High-grade glandular intraepithelial neoplasia
TACG1	G1 Tubular adenocarcinoma
TACG2	G2 Tubular adenocarcinoma
TACG3	G3 Tubular adenocarcinoma, solid variant
PACG1	G1 Papillary adenocarcinoma
PACG2	G2 Papillary adenocarcinoma
MPAC	G3 Micropapillary carcinoma
PCC	Poorly-cohesive cell carcinoma, signet ring cell subtype
PCC-NOS	Poorly-cohesive cell carcinoma, non-signet-ring cell subtype
MAC1	Mucinous adenocarcinoma, type I
MAC2	Mucinous adenocarcinoma, type II
ACLS	Adenocarcinoma with lymphoid stroma
HAC	Hepatoid adenocarcinoma
ACFG	Adenocarcinoma of the fundic glands
SCC	Squamous cell carcinoma of the stomach
NDC	Undifferentiated carcinoma
NED	Neuroendocrine differentiation

Table S2 – Distribution of diagnoses of patients included in the training set for gastric biopsies

ICD-10	Quantity	%
C16.9	472	49%
D00.2	40	4%
D13.1	42	4%
K29.7	416	43%
Total	970	100%

Table S3 – Distribution of patients from the training set with gastric biopsies by gender

ICD-10 code	Gender		Grand total
	Female	Male	
C16.9	232	240	472
D00.2	20	20	40
D13.1	24	18	42
K29.7	306	110	416
Total	582	388	970

Table S4 – Distribution of patients by age in five-year periods

Age	ICD-10 code				Total
	C16.9	D00.2	D13.1	K29.7	
<30 years	-	-	-	16	16
31-35 years	3	-	1	18	22
36-40 years	6	-	1	27	34
41-45 years	12	-	2	36	50
46-50 years	6	1	3	39	49
51-55 years	21	1	4	38	64
56-60 years	48	7	6	60	121
61-65 years	67	6	6	72	151
66-70 years	123	2	9	49	183
71-75 years	72	8	7	35	122
76-80 years	54	4	2	18	78
81-85 years	48	7	1	6	62
>86 years	12	4	-	2	18
Total	472	40	42	416	970

Table S5 – Distribution of patients from the training set with gastric biopsies by age

ICD-10 code	Average age, women	Average age, men	Minimum age, women	Minimum age, male	Maximum age, women	Maximum age, male
C16.9	69	67	38	33	88	88
D00.2	77	65	57	48	89	83
D13.1	67	65	44	57	86	77
K29.7	56	56	18	20	93	83
Total	59	61	18	20	93	88

Table S6 – Distribution of biopsies with gastric carcinomas by ICD-10 codes

ICD-10 code	Quantity	Percentage, %
C16.0	60	13%
C16.1	11	2%
C16.2	128	27%
C16.3	187	40%
C16.4	10	2%
C16.5	14	3%
C16.6	9	2%
C16.8	53	11%
Total	472	100%

Table S7 – Characteristics of gastric biopsies with non-neoplastic pathology

K29.7	Atrophy	Intestinal metaplasia	H. pylori	Inflammation activity
Presence	262 (63%)	101 (24%)	108 (26%)	84 (20%)
Absence	154 (37%)	315 (76%)	308 (74%)	332 (80%)
Total	416	416	416	416

Table S8 – Distribution of surgically treated patients by stage and type of surgery

Stage	Operation			Total
	Submucosal dissection (ESD)	Gastric resection	Gastrectomy	
Stage I	58	64	25	147
Stage II	-	38	14	52
Stage III	-	20	9	29
Total	58	122	48	228

Table S9 – Changes in clinical stage among surgically treated patients based on pathological analysis of surgical specimens

Preoperative stage cTNM	Postoperative stage pTNM			Total
	I	II	III	
I	98	-	-	98
II	34	15	-	49
III	15	37	29	81
Total	147	52	29	228

Table S10 – Distribution of patients by level of tumor regression grade (TRG)

Level regression	Characteristic	n	%
Without neoadjuvant	-	151	-
TRG1	Absence of viable tumor cells or small groups of tumor cells	5	2.2
TRG2	The proportion of fibrosis is greater than the proportion of residual tumor	41	18
TRG3	Residual tumor predominates over fibrosis or no regression	31	13.6
	Total	228	100

Table S11 – Distribution by stages in all groups of patients by treatment

Number of patients by stage	No Surgery	Surgery + neoadjuvant	Surgery without neoadjuvant
IA	4	15	89
IB	6	16	24
IIA	10	20	3
IIB	20	11	22
IIIA	39	6	7
IIIB	4	7	2
IIIC	0	2	4
IV	161	0	0
Total	244	77	151

Table S12 – Her2 status depending on the histological type of gastric cancer

Lauren 's histotype	Histotype according to WHO	Negative (0)	Negative (1+)	Positive (3+/FISH+)	Total
Intestinal	TAC-LG	3	3	4	10
	PAC	3	-	1	4
Diffuse	PCC	7	1	-	8
	PCC-NOS	4	3	-	7
Indeterminate	TAC-HG	6	7	4	17
	MAC	2	-	-	2
Mixed	Mixed	1	-	1	2
Total		26	14	10	50

Table S13 – Serum levels of CEA, CA19-9, and hemoglobin, as well as BMI, according to disease stage

Prevalence of stomach cancer:	Localized process	Regional process	Generalized (Distant/Metastatic) Process	Total
**Carcinoembryonic antigen (CEA):** reference values 0–3.8 ng /ml				
N/A	52	24	201	277
Normal level	52	52	48	152
Increased	4	6	33	43
**CA 19-9:** reference values: 0–34 U /ml.				
N/A	55	27	223	305
Normal level	49	52	46	147
Increased	4	3	13	20
**Blood hemoglobin: anemia in reference values** <130 g/ dL for men, <120 g/ dL for women				
N/A	9	5	186	200
Normal level	57	54	53	164
Anemia	42	23	43	108
**Body mass index (BMI)** at initial examination				
N/A	23	48	240	311
Normal level (18.5-25)	30	2	23	55
Overweight (25-30)	50	13	10	73
Obesity 1 Stage (30-35)	5	19	9	33
Total	108	82	282	472

Table S14 – PD-L1 status depending on the histological type of gastric cancer

Lauren 's histotype	Histotype according to WHO	CPS = 0	CPS ≥ 1	Total
Intestinal	PAC	3	2	5
	TAC-LG	4	5	9
Diffuse	PCC	7	-	7
	PCC-NOS	1	6	7
Indeterminate	TAC-HG	10	7	17
	MAC	3	-	3
Mixed	Mixed	1	1	2
Total		29	21	50

Figure S1 – Histotype in the initial biopsy study before annotation

**Histotype**	**Quantity**	**%**
AC	6	1%
AC-LG	57	12%
AC-HG	60	13%
PAC	5	1%
PAC-LG	18	4%
PCC	75	22%
PCC-NOS	35	1%
TAC-HG	90	19%
TAC-LG	117	25%
Mixed	5	1%
NEC	4	1%
**Total**	472	100%

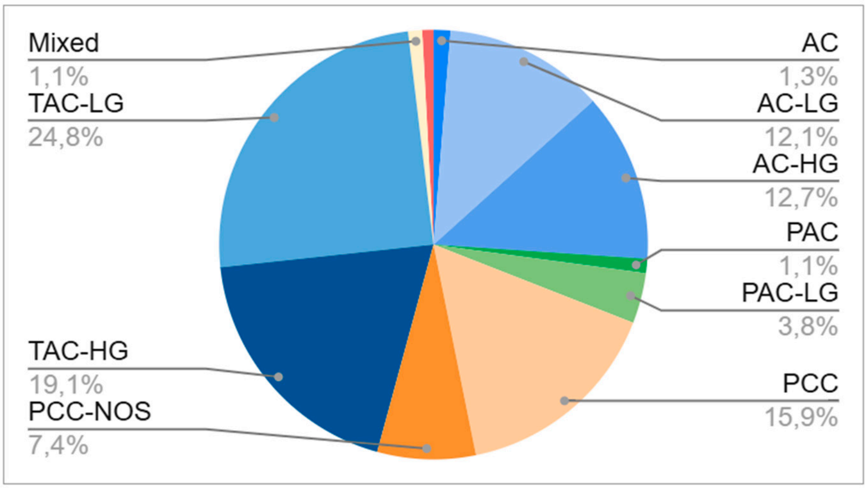

Figure S2 – Foci of intestinal metaplasia. Hematoxylin and eosin staining. Magnification x300

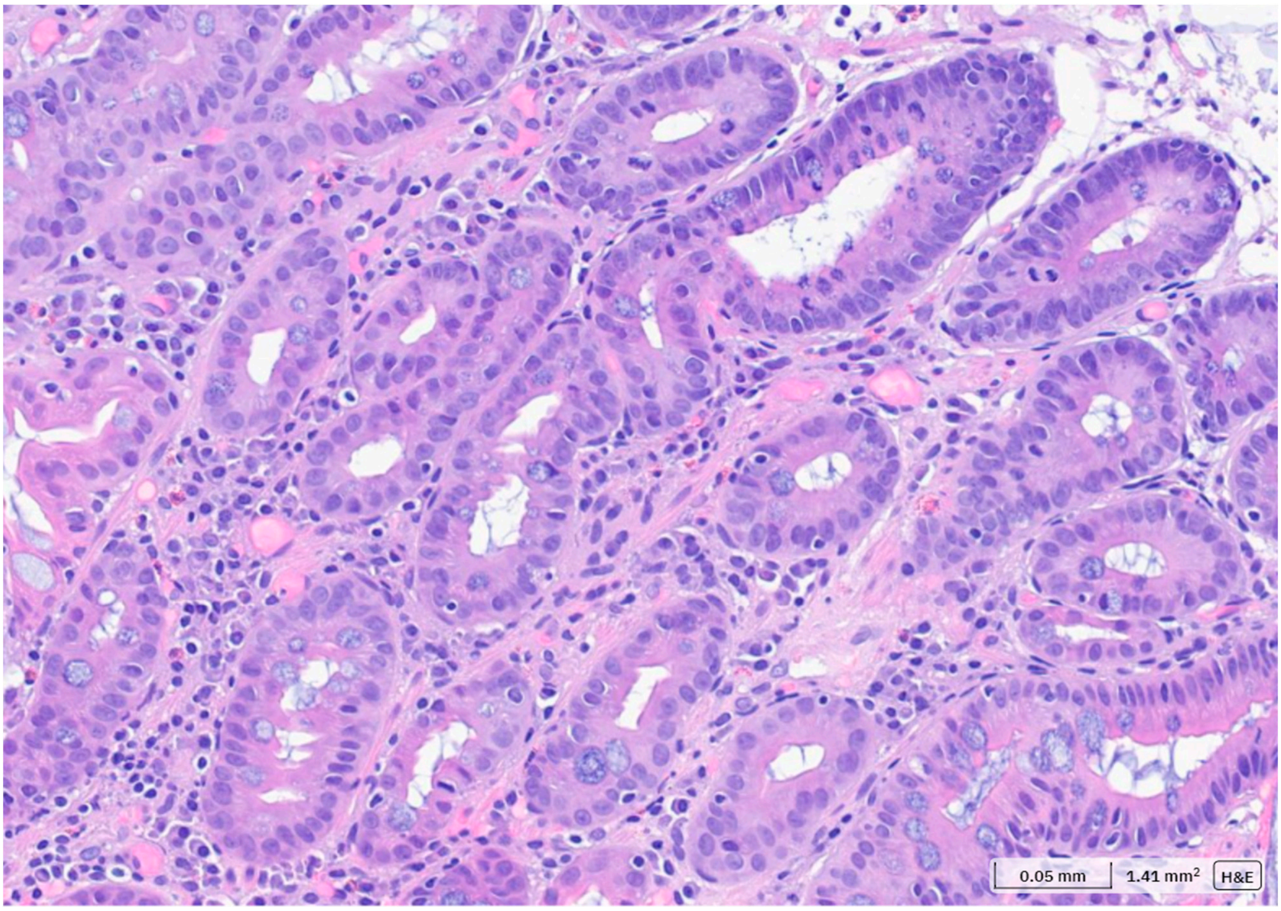

Figure S3 – Examples of slices with the annotation “Normal glands”

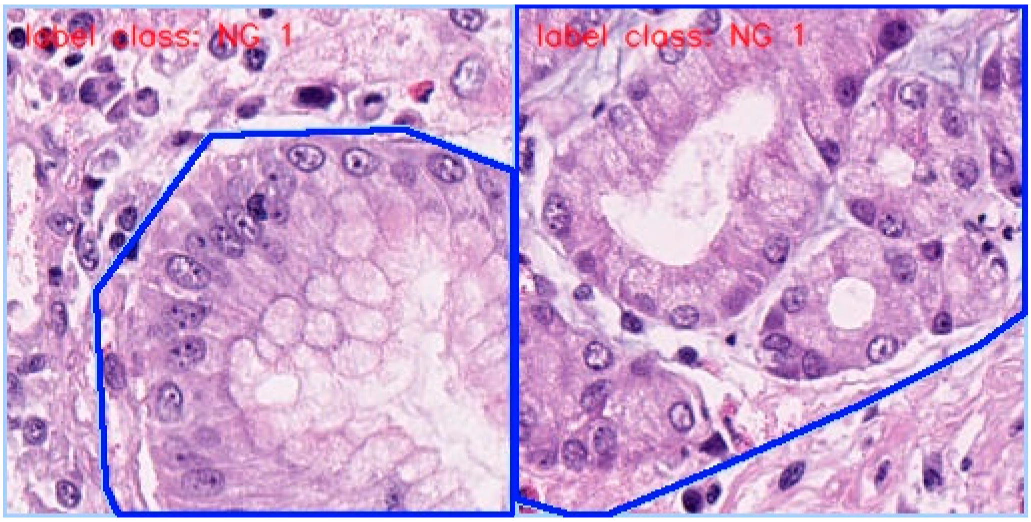

Figure S4 – Example of a slice with a zone of interest area less than 90%

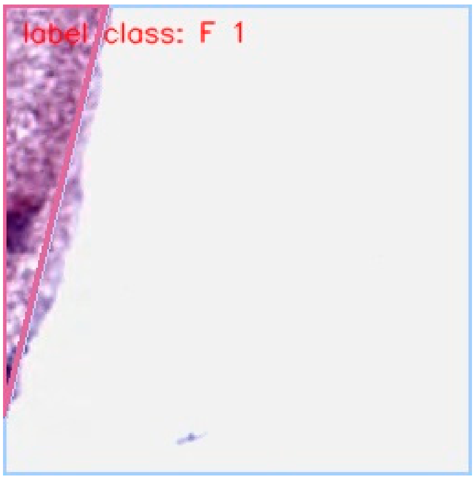

Figure S5 – An example of a slice that does not contain the declared annotated region

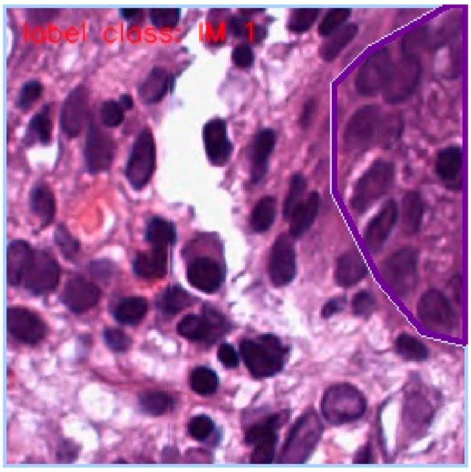

Table S15 – Total area of annotation according to ICD-10 codes in pixels

**Class / area, pixels**	Total area of biopsies	Total area of annotation	Total area of tumor	Total area of dysplasia Low and High	Total area of annotation non-tumor tissue
C16.9	39 390 463 186	35 809 511 982	20 709 875 415	1,175,304,982	13 924 331 585
D00.2	2 425 218 264	2 309 731 680	-	1 600 886 420	708 845 260
D13.1	3 870 630 435	3 617 411 628	-	345 642 416	3 271 769 212
K29.7	38 389 509 684	34 585 143 872	-	-	34 585 143 872
**Total**	84 075 821 598	76 321 799 162	20 709 875 415	3 121 833 818	52 490 089 929

Table S16 – Total area of annotation for individual histological classes in pixels depending on the ICD-10 code

**Class/area pixels**	C16.9	D00.2	D13.1	K29.7	Total
TAC-LG	6 664 074 401	-	-	-	6 664 074 401
TAC-HG	4 598 290 866	-	-	-	4 598 290 866
NED	144 753 471	-	-	-	144 753 471
PAC-LG	1 817 583 758	-	-	-	1 817 583 758
PCC	4 244 555 130	-	-	-	4 244 555 130
PCC-NOS	2 510 671 578	-	-	-	2 510 671 578
NDC	101,471,957	-	-	-	101,471,957
MAC	543 032 134	-	-	-	543 032 134
MPAC	16 310 441	-	-	-	16 310 441
ACLS	69 131 679	-	-	-	69 131 679
GINH	362 815 897	257 459 100	-	-	620 274 997
GINL	812 489 085	1,343,427,320	345 642 416	-	2 501 558 821
SE (squamous epithelium)	636 083 680	-	-	-	636 083 680
LT (lymphoid tissue)	248 678 683	-	95 068 264	332 723 148	676 470 095
F (fibrin)	1,352,743,048	183 714 420	-	45 502 131	1,581,959,599
IM (intestinal metaplasia)	1,729,528,767	202,130,000	564 493 468	1 876 365 366	4 372 517 601
NG (normal)	9 357 167 782	323,000,840	2 612 207 480	32 268 089 228	44 560 465 330
GT (granulation)	600 129 625	-	-	62,463,999	662 593 624
**Total area of the annotation**	35 809 511 982	2 309 731 680	3 617 411 628	34 585 143 872	76 321 799 162

Table S17 – Frequency of occurrence of various classes by ICD-10 groups in gastric biopsies (according to annotation data)

ICD-10	Frequency of occurrence of different classes (>1% of the biopsy area)						n
	NG	IM	LT	F/GT	GINL	GINH	
C16.9	281	91	75	133	50	40	472
D00.2	22	11	4	15	33	40	40
D13.1	33	14	12	1	42	0	42
K29.7	412	98	137	4	0	0	416
Total	773	265	234	157	132	80	970

Figure S6 – Proportions of annotated classes and subtypes of carcinomas

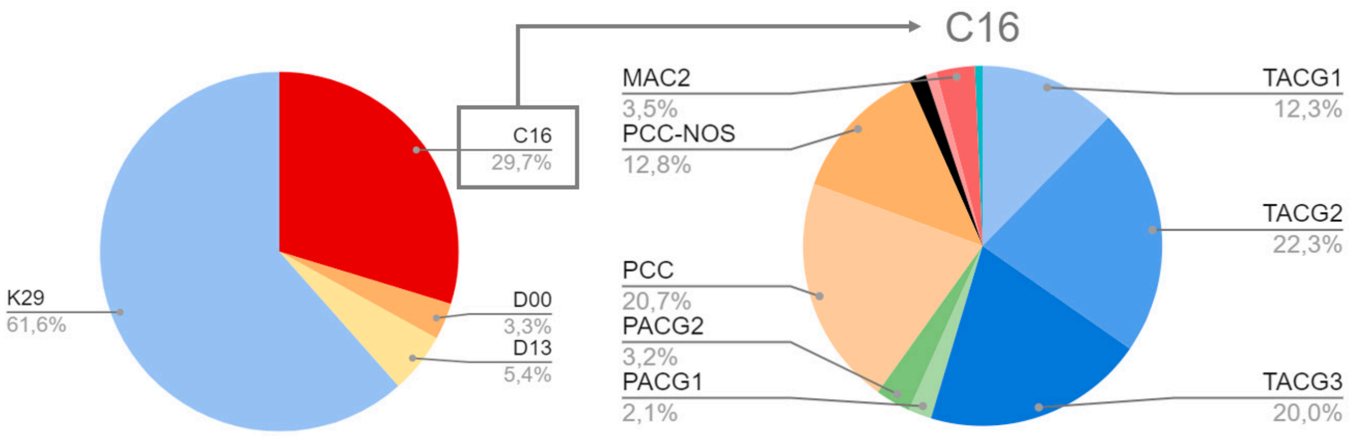

Figure S7 – Histological specimen artifacts. On the left is a manufacturing artifact – folds; on the right is a biopsy artifact (gland fragments, likely tumor-related). Hematoxylin and eosin staining.

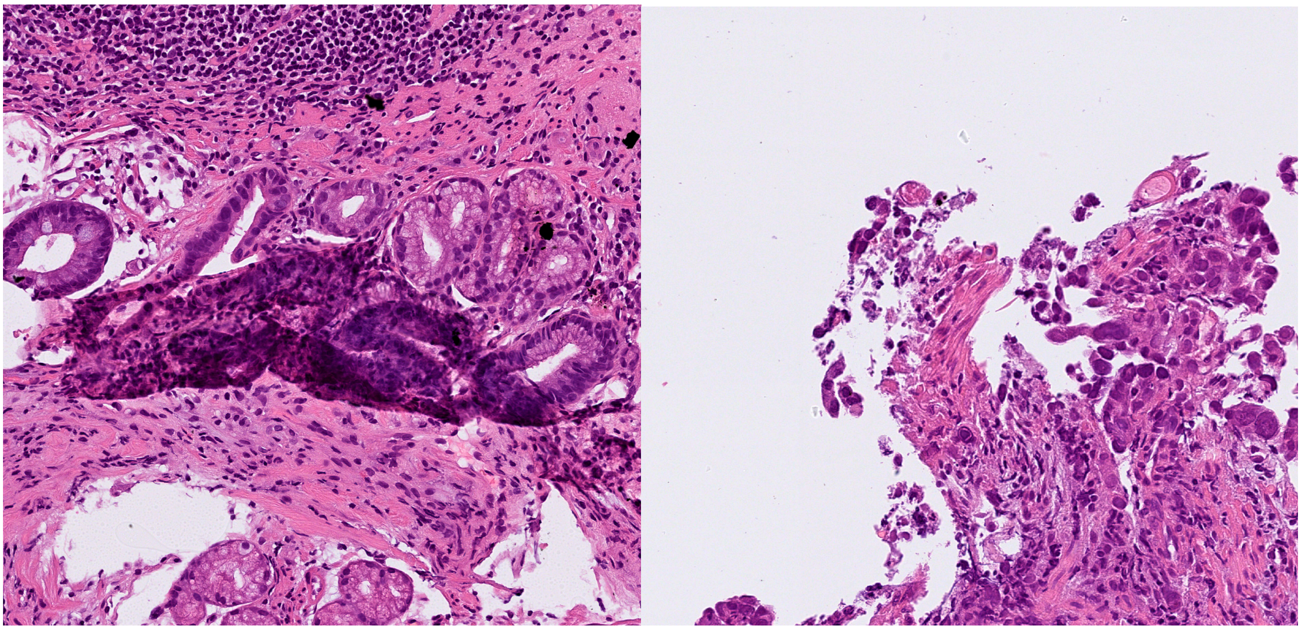

Figure S8 – Changes not included in the annotation. Left – stromal elements, right – peritumoral myxomatosis, which cannot be clearly attributed to tumor growth (carcinoma is circled with a yellow marker; myxomatosis is indicated by a green arrow). Hematoxylin and eosin staining, magnification x100

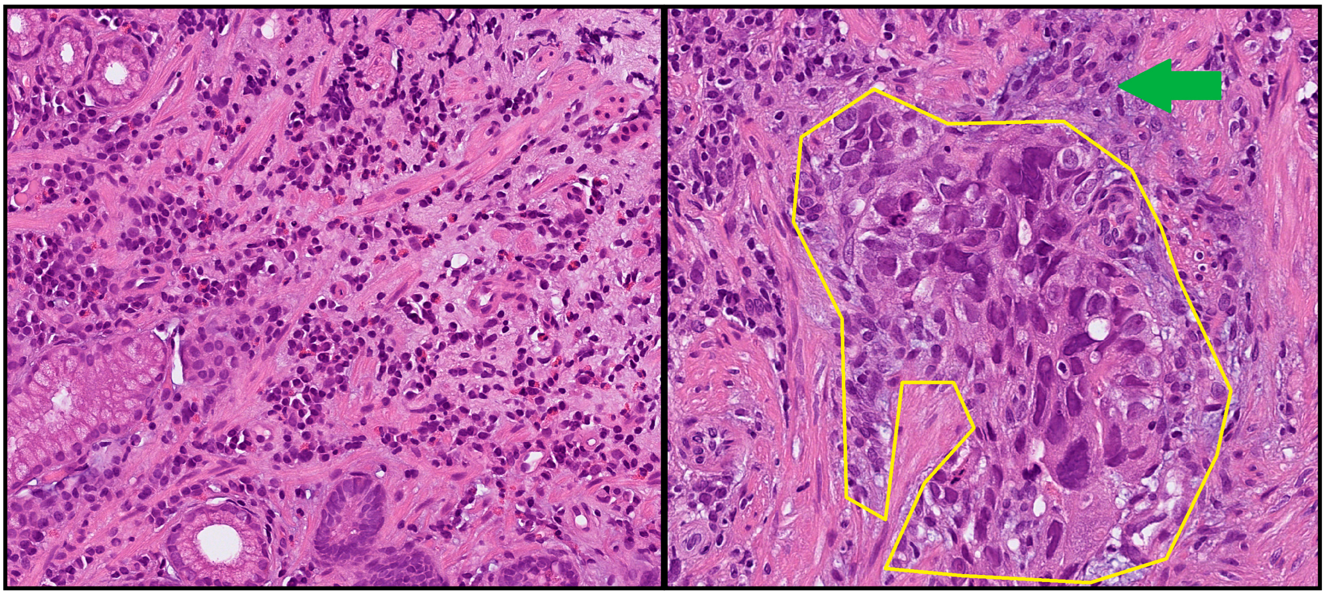

Table S18 – Number of class components when establishing different threshold values at which division into subtypes occurs

Powder value / number of components	Unicomponent tumors	Two-component tumors	Tumors with >2 components
>1% of the tumor area	290	130	52
>10% of the tumor area	343	109	20
>30% of the tumor area	410	62	-

Table S19 – Distribution of carcinomas by number of components

Histotypes with 100% and multicomponent	Quantity
TAC-LG	63
TAC-HG	47
PAC	5
PCC	109
PCC-NOS	50
MAC	8
NDC	3
NEC	5
two-component tumors	130
more than two components	52
Total	472

Figure S9 – Distribution of carcinomas by the number of components (more than 1%)

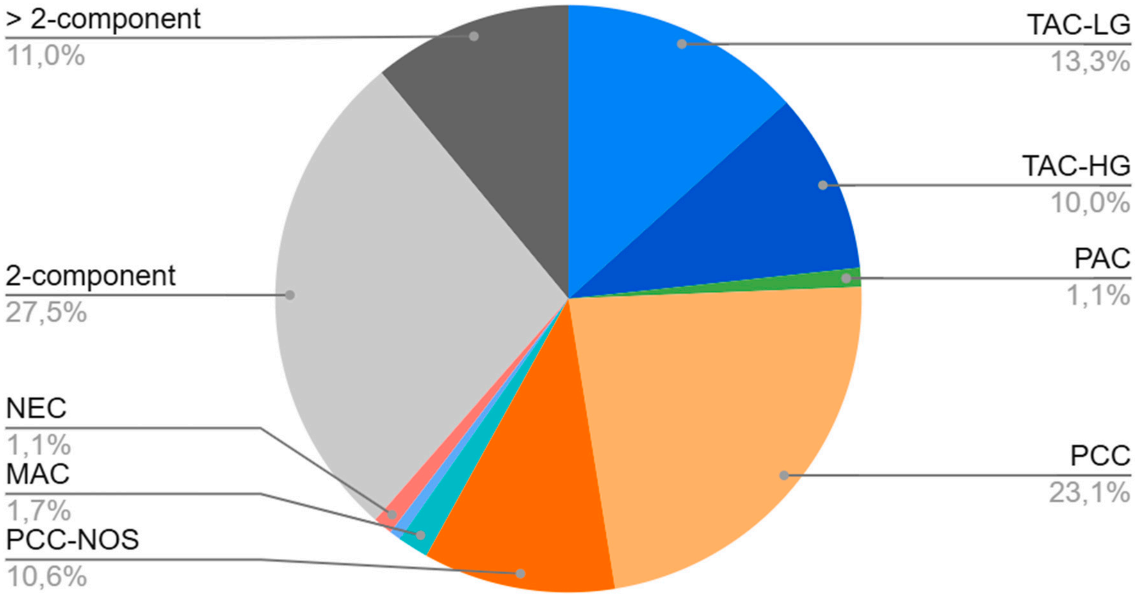

Figure S10 – Distribution of histological types according to the WHO classification after annotation

**Histotype**	**Quantity**
TAC-LG	98
TAC-HG	119
PAC-LG	15
PAC-HG	2
PCC	122
PCC-NOS	63
MAC	18
NEC	7
NDC	5
Mixed	23
**Total**	472

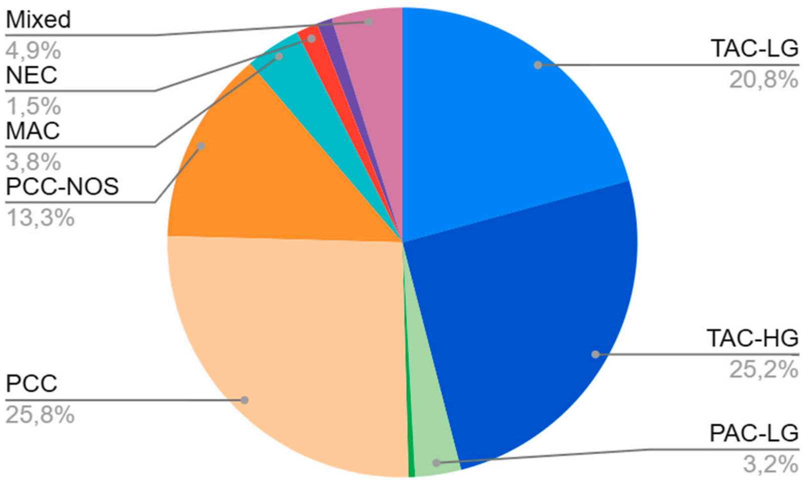

Figure S11 – Left – poorly cohesive cell carcinoma, signet ring cell subtype. Right – poorly cohesive cell carcinoma, non- signet ring cell subtype.

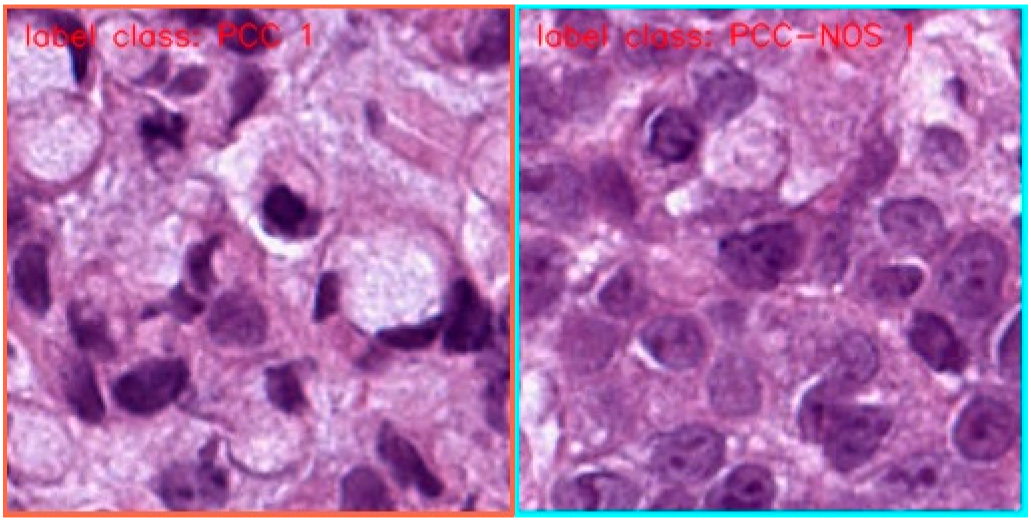

Figure S12 – Distribution of histological types after annotation according to the WHO classification, taking into account the addition of the subtype “poorly cohesive cell carcinoma, combined subtype” according to F. Carneiro

Histotype	Quantity
TAC-LG	98
TAC-HG	119
PAC-LG	15
PAC-HG	2
PCC	111
PCC-NOS	60
**PCC-combined**	**14**
MAC	18
NEC	7
NDC	5
Mixed	23
**Total**	472

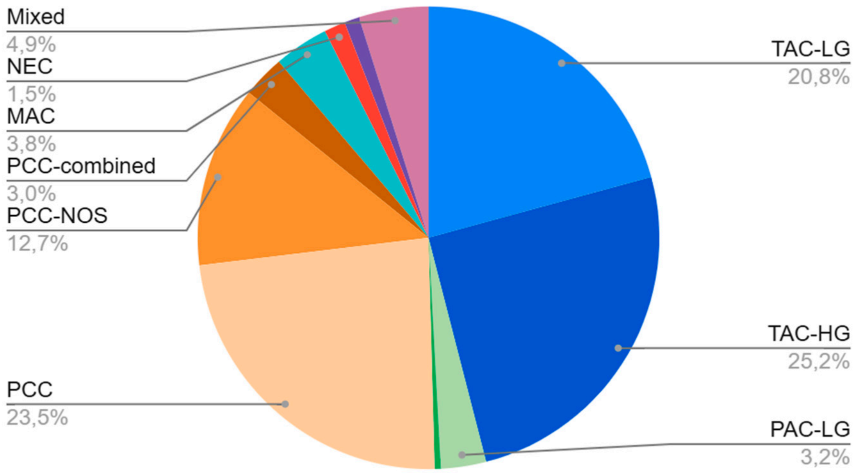

Figure S13 – Examples of slices with labeled structures of high-grade tubular adenocarcinoma . Hematoxylin and eosin staining

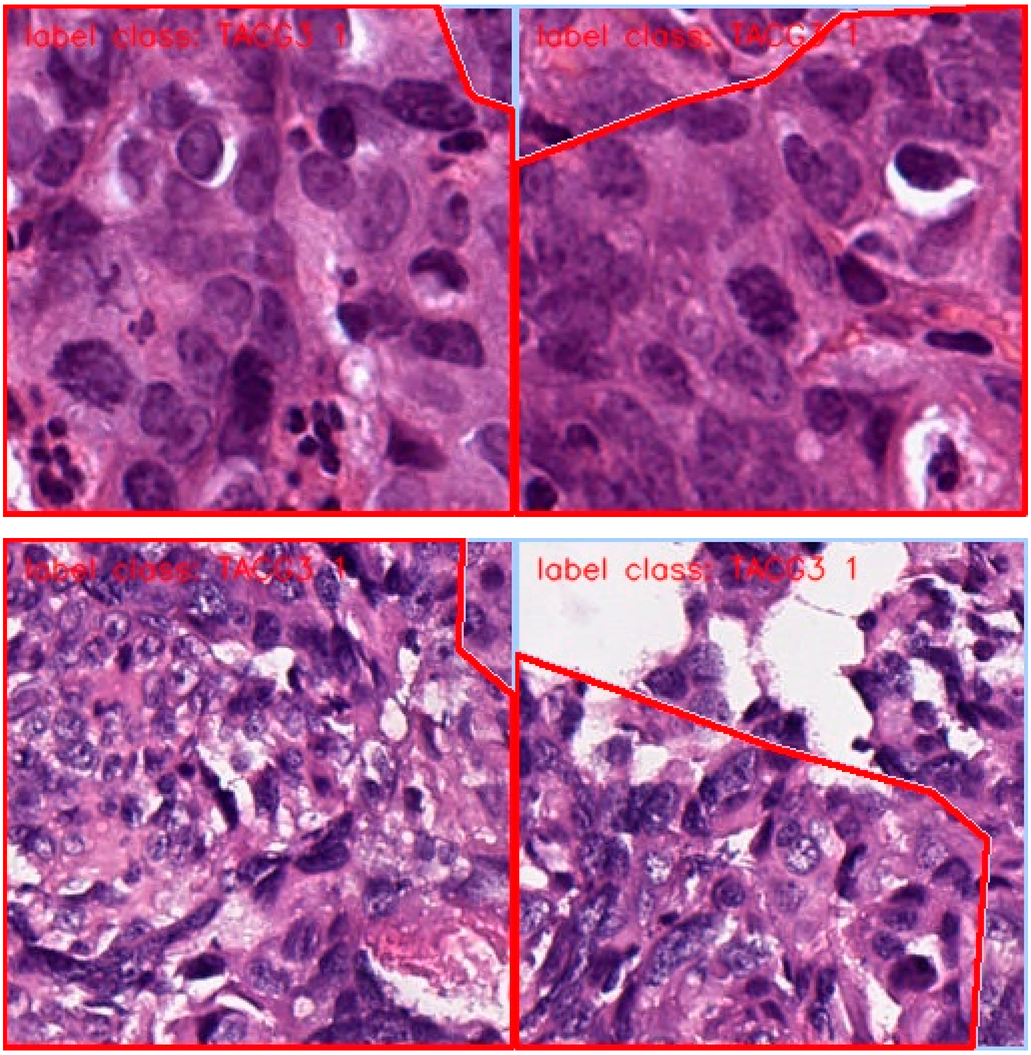

Figure S14 Examples of slices with labeled structures of low-grade papillary adenocarcinoma slices . Left – PACG1. Right – PACG2. Hematoxylin and eosin staining.

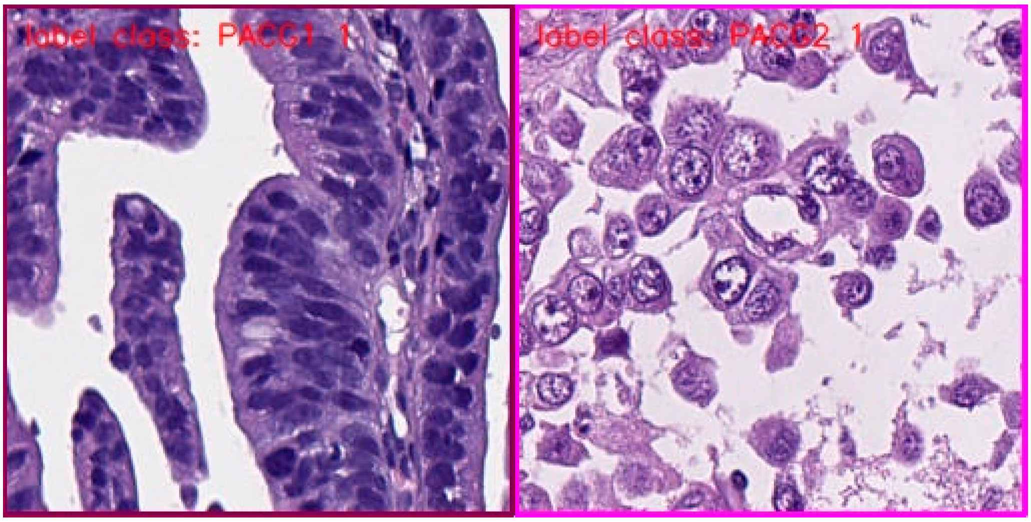

Table S20 – Correspondence between the Lauren classification and the 2019 WHO classifications

Histotype according to Lauren (1965)	histotype (2019)
Intestinal	- Papillary<br>- Tubular, well differentiated<br>- Tubular, moderately differentiated
Indeterminate	Tubular (solid), poorly differentiated
Diffuse	signet-ring cell carcinoma .<br>poorly-cohesive cell carcinoma, other cell types
Intestinal/ diffuse/ unspecified	Mucinous
Mixed	Mixed
Not defined	Other histologic subtypes:<br>Adenosquamous carcinoma<br>Squamous cell carcinoma<br>Undifferentiated cancer<br>Carcinoma with lymphoid stroma<br>Hepatoid carcinoma<br>Adenocarcinoma with enteroblastic differentiation<br>Fundic gland type adenocarcinoma<br>Micropapillary adenocarcinoma

Table S21 – Changes in histological type in gastric biopsies according to the Lauren classification before and after annotation

Lauren type primary (below)	Lauren histotype after annotation				Total
	Diffuse	Indeterminate	Intestinal	Mixed	
Diffuse	108	1	-	1	110
Indeterminate	43	88	12	11	154
Intestinal	34	62	101	6	203
Mixed	-	-	-	5	5
Total	185	151	113	23	472

Table S22 – Change in grade in gastric biopsies before and after annotation

Grade primary	Grade after annotation			Total
	G1	G2	G3	
Not specified	0	3	7	10
G1	15	30	15	60
G2	6	42	84	132
G3	0	17	253	270
Total	21	92	359	472

Table S23 – Proportion of all components in adenocarcinomas with mixed subtypes

Histotype according to WHO	Histotype: dominant	Grade	Histological patterns (% of total carcinoma annotation area)								
			TAC-LG	TAC-HG	PAC	MPAC	PCC	PCC-NOS	NED	NDC	MAC
Mixed	PAC	High	20	31	49	-	-	-	-	-	-
Mixed	MAC	High	9	9	38	-	-	-	-	-	43
Mixed	NED	High	-	43	-	-	-	-	57	-	-
Mixed	PAC	High	1	32	57	10	-	-	-	-	-
Mixed	PCC	High	11	29	-	-	-	59	-	-	-
Mixed	TAC-LG	Low	57	-	43	-	-	-	-	-	-
Mixed	PCC	High	43	-	-	-	-	57	-	-	-
Mixed	TAC-HG	High	-	54	46	-	-	-	-	-	-
Mixed	PCC	High	11	29	-	-	-	59	-	-	-
Mixed	MAC	High	27	-	9	-	15	-	-	-	49
Mixed	PCC	High	-	44	-	-	56	-	-	-	-
Mixed	TAC-HG	High	-	57	-	-	43	-	-	-	-
Mixed	PCC	High	-	-	-	-	51	-	-	-	49
Mixed	TAC-HG	High	14	44	42	-	-	-	-	-	-
Mixed	TAC-LG	High	52	-	-	-	-	-	-	-	48
Mixed	TAC-LG	Low	53	-	47	-	-	-	-	-	-
Mixed	PAC	Low	45	-	55	-	-	-	-	-	-
Mixed	TAC-LG	High	53	-	-	-	-	-	-	47	-
Mixed	NDC	High	44	-	-	-	-	-	-	56	-
Mixed	TAC-LG	High	54	-	-	-	-	-	-	-	46
Mixed	TAC-LG	High	51	-	-	-	-	49	-	-	-
Mixed	PCC	High	34	-	-	-	-	52	-	-	14
Mixed	MAC	High	5	5	-	-	45	-	-	-	45

Figure S15 – Comparison of histotypes according to WHO and the dominant component

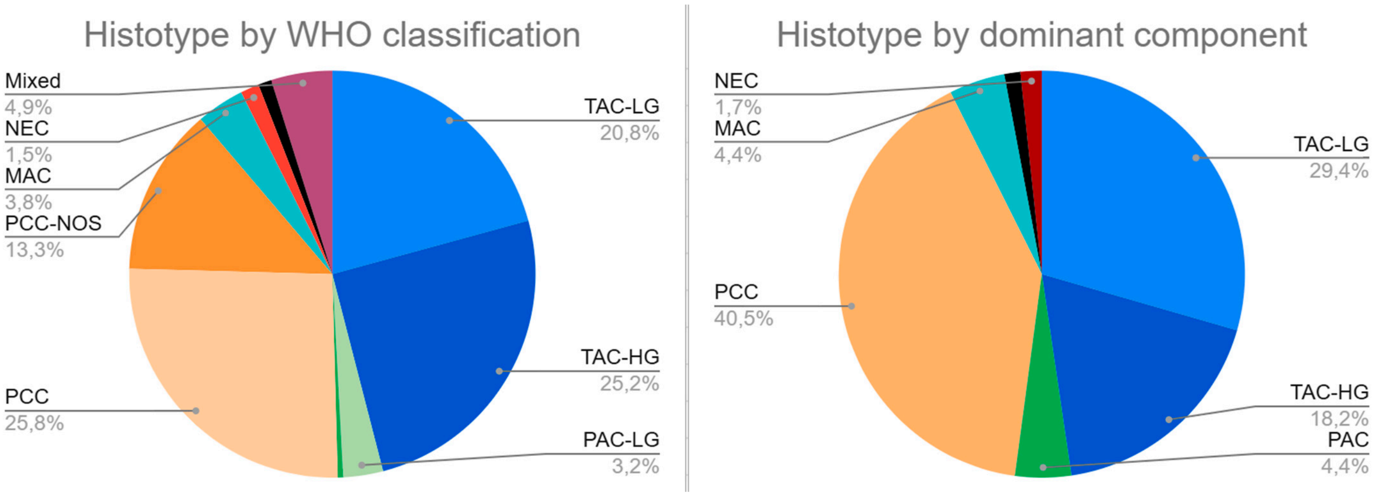

Figure S16 – Diagram of survival curves for patients with gastric carcinoma depending on age (older/younger than 65 years)

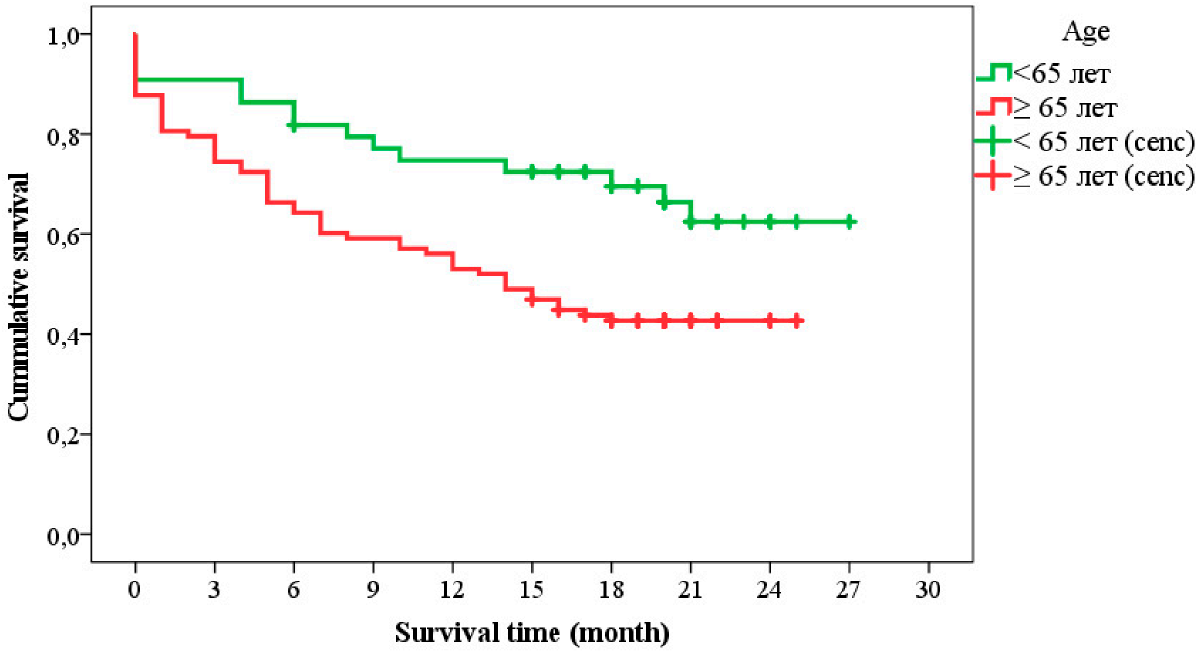

Figure S17– Kaplan–Meier survival curves for patients with gastric carcinoma depending on the TNM-stage

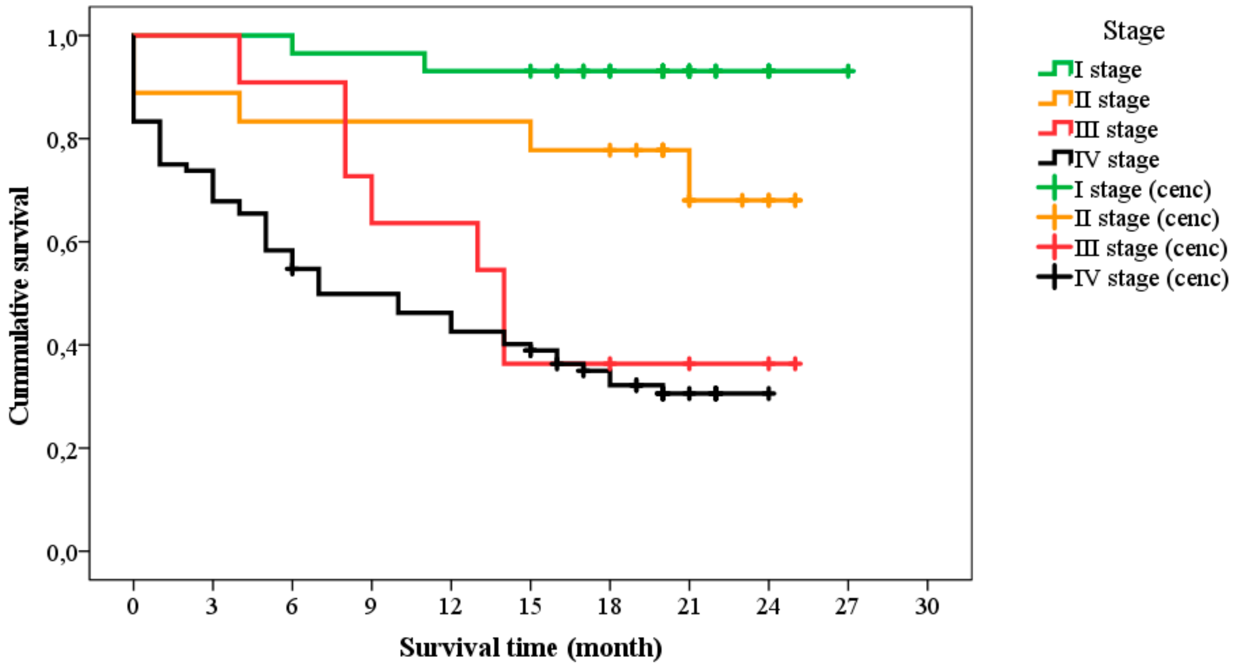

Figure S18 – Kaplan–Meier survival curves of gastric cancer patients stratified into three groups according to the extent of disease, following the SEER

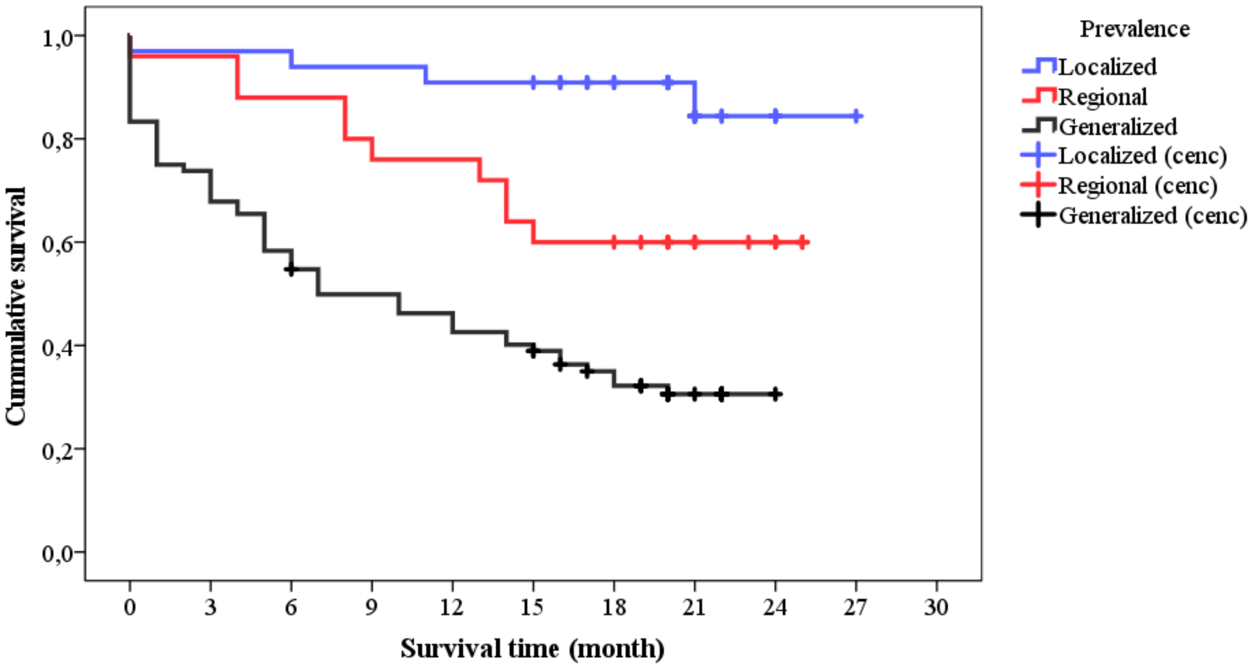

Figure S19 – Kaplan–Meier survival curves for patients with gastric carcinoma depending on the established subtype according to the Lauren classification in the primary study (before annotation)

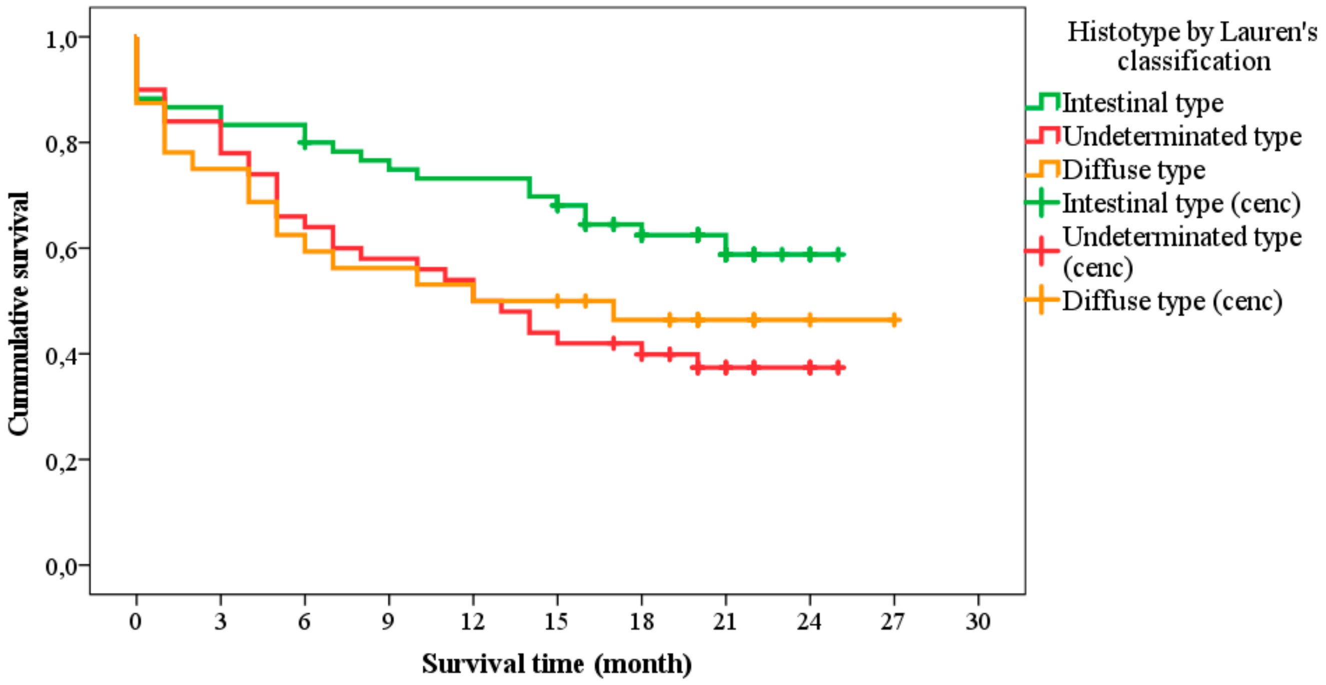

Figure S20 – Kaplan–Meier survival curves for patients with gastric carcinoma depending on the degree of differentiation according to the two-level grading system (Low /High- Grade) during the initial examination (before annotation)

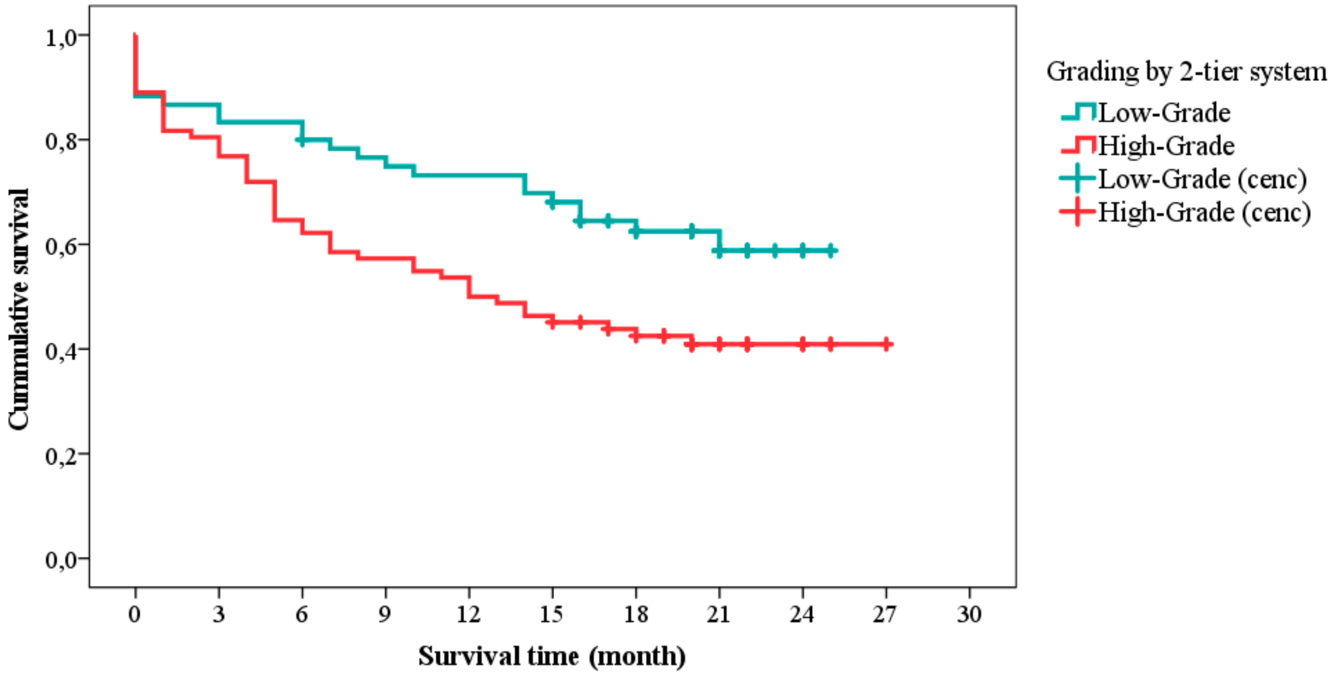

Figure S21 – Kaplan–Meier survival curves for patients with gastric carcinoma depending on the degree of differentiation according to the three-level grading system (G1, G2, G3) during the initial examination (before annotation)

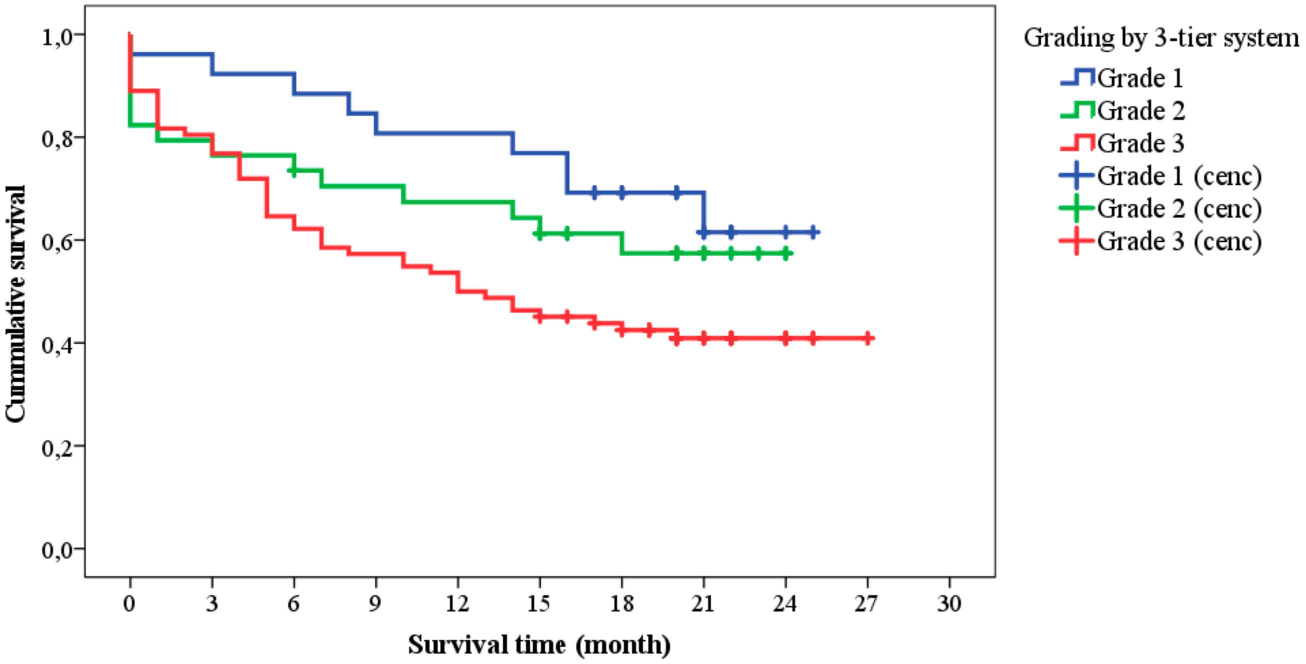

Figure S22 – Kaplan–Meier survival curves of histological types according to the association of tumor with intestinal metaplasia according to the annotation data

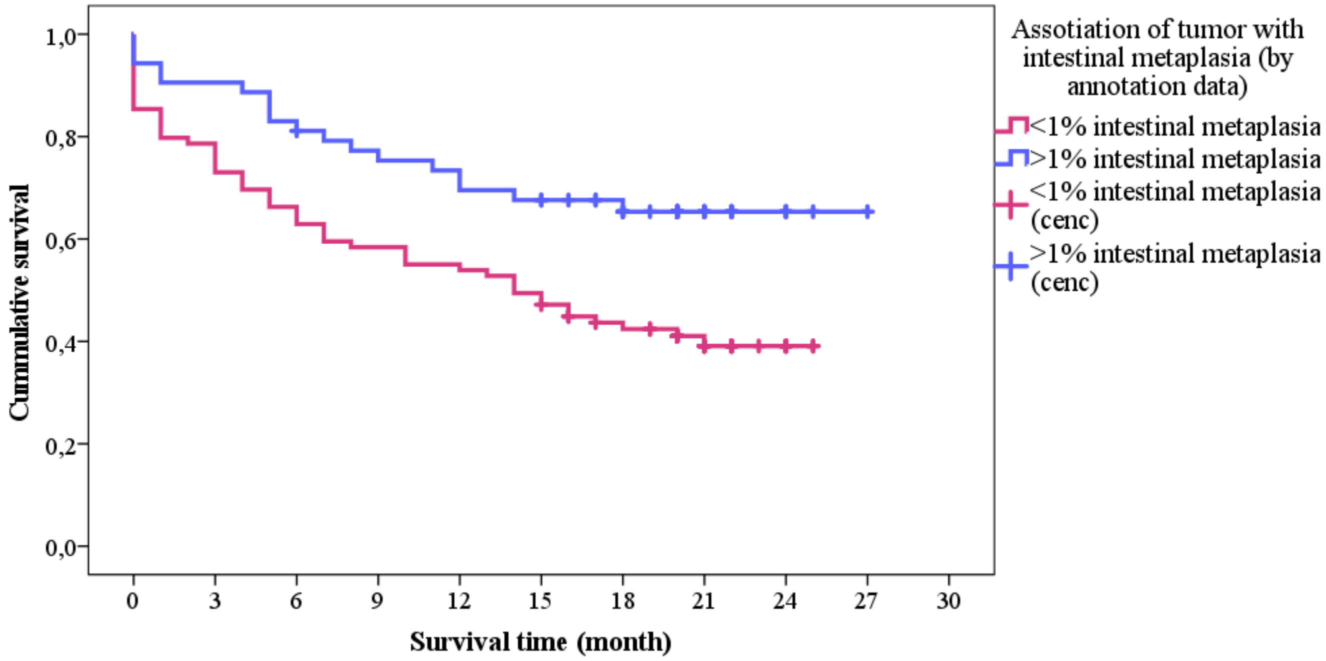

Figure S23 – Kaplan–Meier survival curves for patients with gastric tumor with and without ulceration

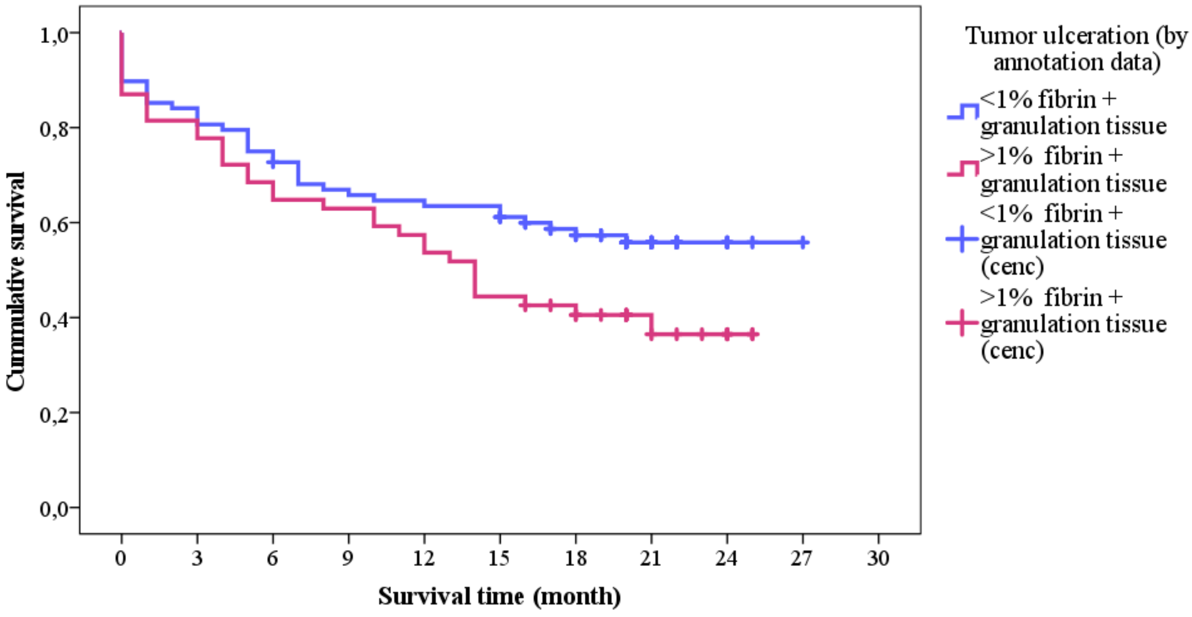

Figure S24 – Kaplan–Meier survival curves for patients whose biopsies contain only tumor tissue and those whose biopsy specimens contain gastric mucosa in addition to the tumor

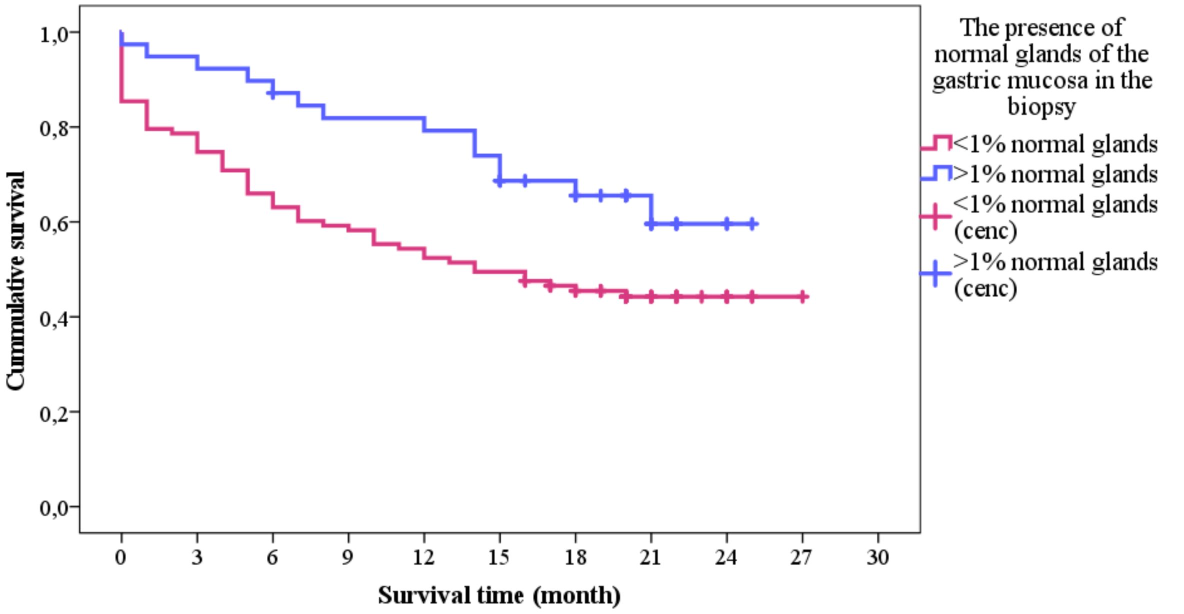

Figure S25 – Kaplan–Meier survival curves for patients in whose biopsies the tumor developed against the background of dysplastic changes and those in whom dysplastic changes were not detected in the biopsy

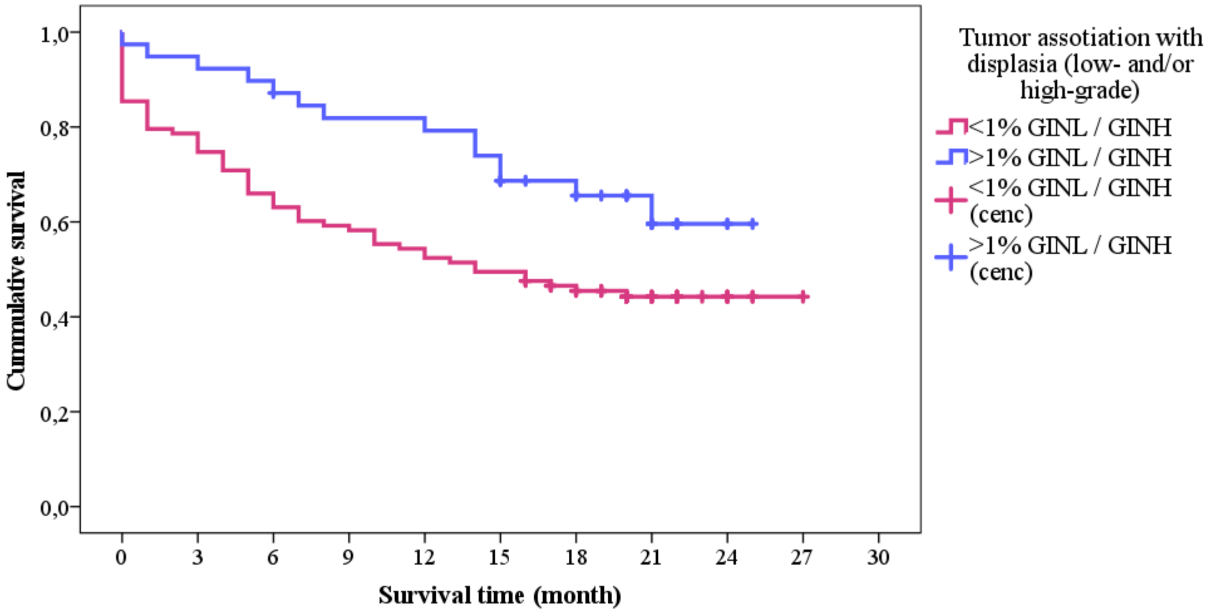

Table S24 – Comparison of cut-off values for determining the low-grade component in carcinoma with overall survival (OS)

Clinical and pathological parameters	Total patients (n = 472)	Group with OS (n = 142)	Median survival time (months, 95% CI)	One-year survival rate (%)	Log - rank	p - value
**Comparison of groups when setting a threshold of 1% HG component**						
<1% of the HG	122	37	17.9 (14.8 - 20.7)	76	1,410	0.235
>1% of the HG	350	105	15.7 (13.5 - 17.9)	54		
**Comparison of groups when setting a threshold of 10% HG component**						
<10% of the HG	134	41	17.9 (15.0 - 20.7)	74	2,159	0.142
>10% of the HG	338	99	1 5.5 (13.3 - 17.8)	54		
**Comparison of groups when setting a threshold of 20% HG component**						
<20% of the HG	142	49	17.4 (14.7 - 20.1)	71	1,917	0.166
>20% of the HG	330	93	15.5 (13.2 - 17.8)	54		
**Comparison of groups when setting a threshold of 30% HG component**						
<30% of the HG	153	51	17.6 (15.0 - 20.2)	72	3,170	0.075
>30% of the HG	319	87	15.0 (12.6 - 17.4)	52		
**Comparison of groups when setting a threshold of 40% HG component**						
<40% of the HG	155	58	17.5 (15.0 - 20.0)	71	3,124	0.077
>40% of the HG	317	84	15.9 (12.6 - 17.5)	52		
**Comparison of groups when setting the threshold at 50% HG component**						
<50% of the HG	160	59	17.5 (15.0 - 19.9)	71	2,824	0, 093
>50% of the HG	312	83	15.0 (12.6 - 17.5)	52		
**Comparison of groups when setting the threshold at 60% HG component**						
<60% of the HG	168	61	17.5 (15.1 - 19.9)	70	2,940	0.086
>60% of the HG	304	81	14.9 (12.5 - 17.5)	52		
**Comparison of groups when setting the threshold at 70% HG component**						
<70% of the HG	176	65	17.6 (15.3 - 19.9)	**71**	**4,050**	**0.044**
>70% of the HG	296	77	14.7 (12.1 - 17.2)	**51**		
**Comparison of groups when setting the threshold at 80% HG component**						
<80% of the HG	186	71	17.2 (14.9 - 19.5)	69	2,914	0.088
>80% of the HG	286	71	14.7 (12.1 - 17.4)	51		
**Comparison of groups when setting the threshold at 90% HG component**						
<90% of the HG	195	74	16.5 (14.3 - 18.8)	66	1,208	0.272
>90% of the HG	277	68	15.3 (12.6 - 18.0)	53		
**Comparison of groups when setting the threshold at 99% HG component**						
<99% HG	205	80	16.2 (13.9 - 18.3)	64	0,507	0.476
>99% of the HG	267	62	15.7 (12.9 - 18.5)	55		

Figure S26 – Comparison of the thresholds for determining the degree of differentiation depending on the sum of the % of the high-grade component in the tumor, using the Kaplan-Meier survival analysis method

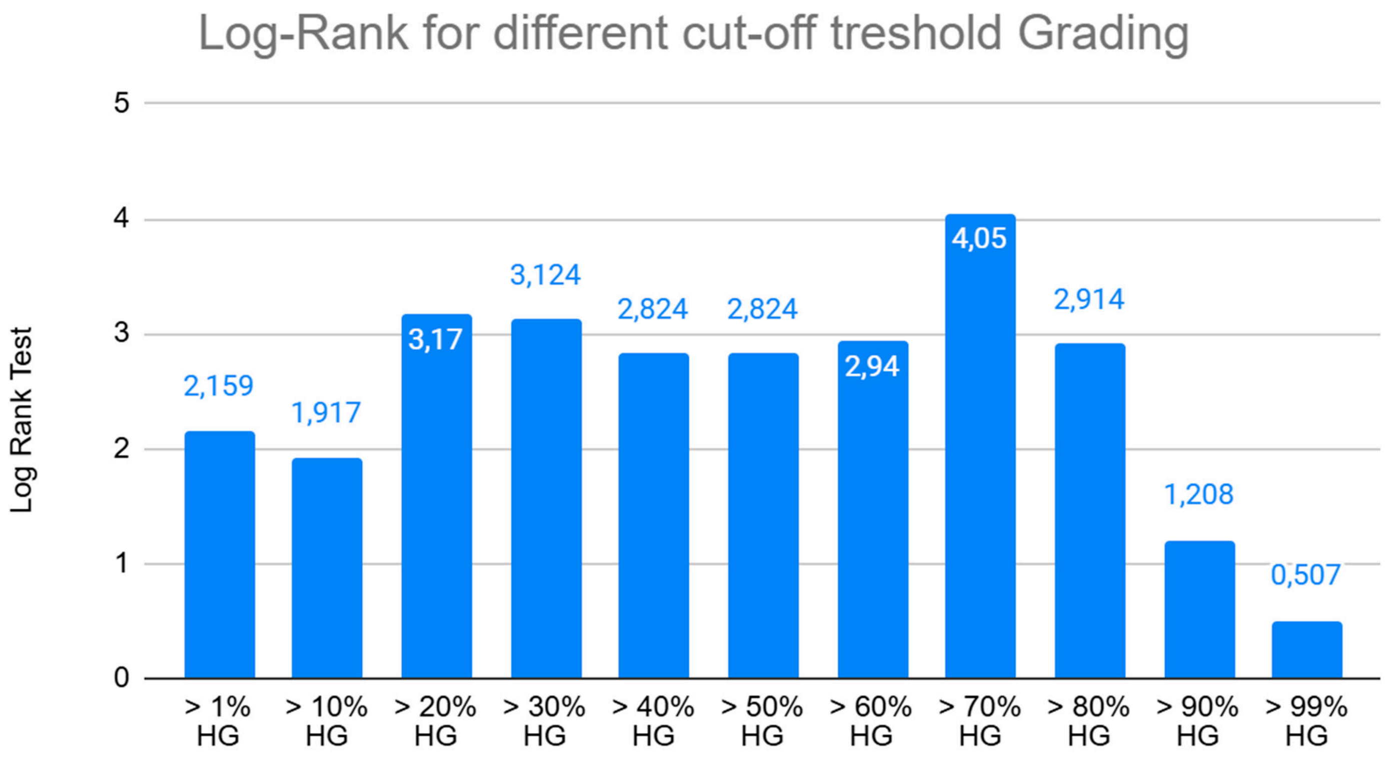

Table S25 – Distribution of histological types in the initial study, threshold value of high-grade component >1% and more than >70%

Distribution by histotypes, quantity	Before annotation	After annotation with the HG threshold of 1%	After annotation with the HG threshold of 70%
AC-NOS	5	0	0
AC-LG	57	0	0
AC-HG	61	0	0
TAC-LG	117	98	143
TAC-HG	90	119	79
PAC	5	0	0
PAC-LG	18	15	18
PAC-HG	0	2	0
PCC	75	122	119
PCC-NOS	35	63	65
MAC	0	18	15
NDC	0	5	4
NEC	4	7	8
Mixed	5	23	21
Total	472	472	472

Figure S27 – Diagrams of changes in histological type before annotation, after annotation by 1% and after changing the threshold value by 70%

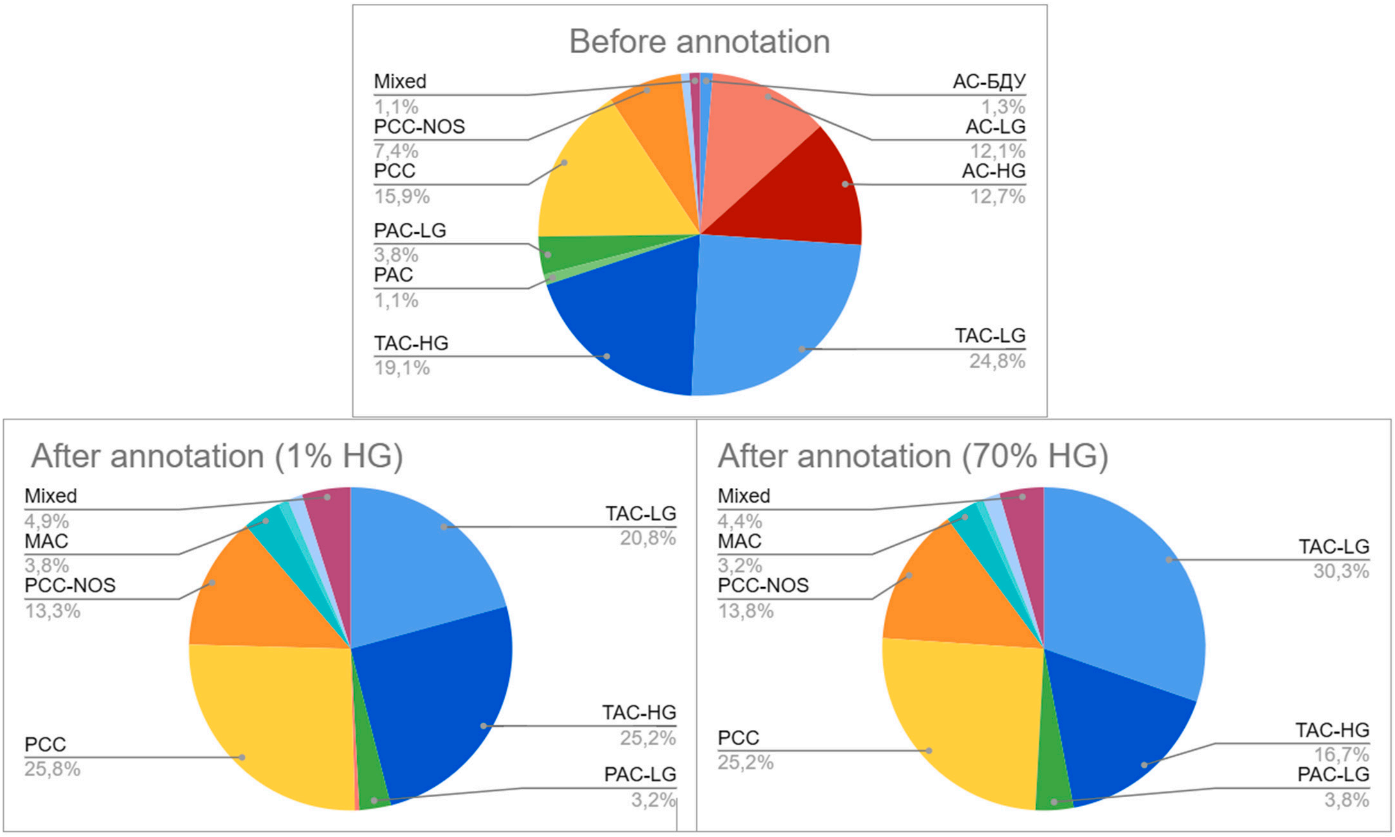

Figure S28 – Kaplan–Meier survival curves depending on the degree of differentiation (Grade) after annotation (cut-off 70% HG)

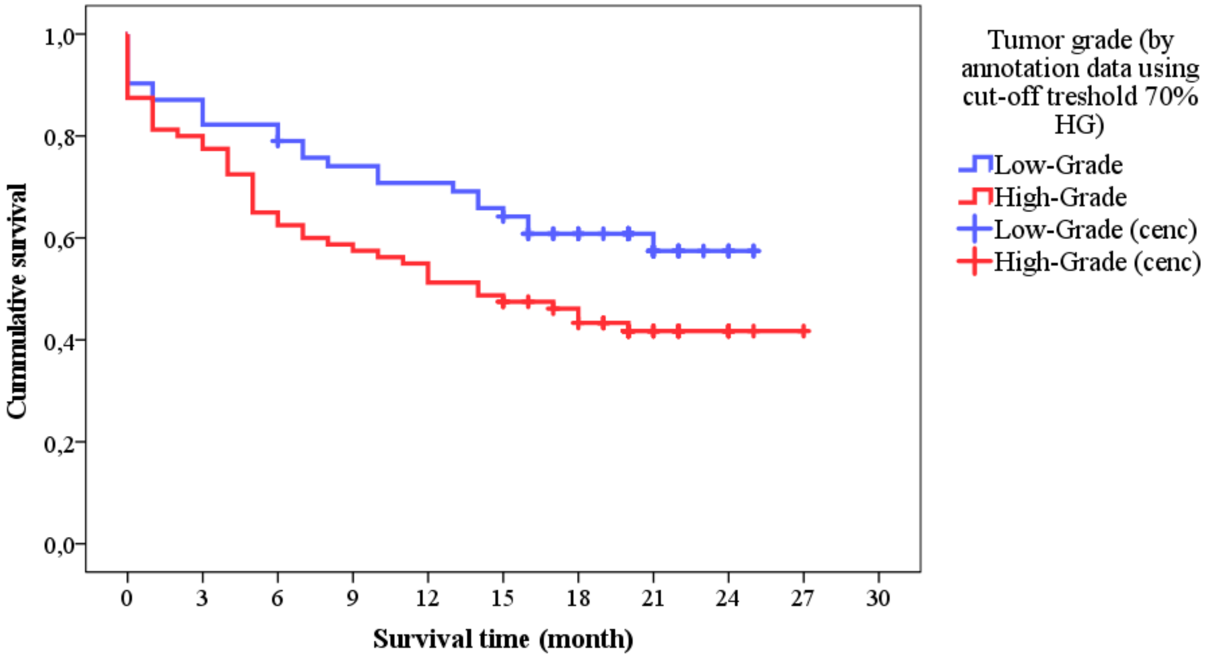

Figure S29 – Correlation matrix of clinicopathological parameters; cell values are presented in the following format: Pearson Chi-square coefficient, statistical significance (p).

	AGE	GEN	STAGE	Lauren1	Grade1	Lauren2	Grade2	GIN-L/H	IM	F	HER2	PD-L1
AGE		0,947 p=0,33	1,327 p=0,51	0,361 p=0,835	0,053 p=0,819	3,625 p=0,163	0,199 p=0,656	1,031 p=0,31	0,091 p=0,763	0,445 p=0,505	0,952 p=0,329	1,523 p=0,218
GEN			5,048 p=0,08	1,132 p=0,568	1,125 p=0,289	0,282 p=0,869	0,154 p=0,695	0,167 p=0,683	9,567 p<0,001	1,099 p=0,295	2,914 p=0,088	0,026 p=0,871
STAGE				10,323 p=0,035	8,621 p=0,013	9,055 p=0,06	6,606 p=0,037	5,264 p=0,072	4,927 p=0,085	2,591 p=0,274	2,058 p=0,357	8,373 p=0,015
Lauren1					157,313 p<0,001	151,199 p<0,001	98,649 p<0,001	7,325 p=0,026	2,874 p=0,238	2,286 p=0,319	5,164 p=0,076	8,225 p=0,016
Grade1						96,309 p<0,001	95,691 p<0,001	4,906 p=0,027	1,521 p=0,218	0,748 p=0,387	3,934 p=0,047	0,086 p=0,77
Lauren2							458,896 p<0,001	27,491 p<0,001	12,051 p<0,001	3,521 p=0,171	7,692 p=0,021	0,451 p=0,798
Grade2								27,838 p<0,001	4,601 p=0,032	0,436 p=0,509	5,466 p=0,019	0,399 p=0,528
GIN-L/H									34,113 p<0,001	0,163 p=0,687	0,262 p=0,609	0,402 p=0,526
IM										3,389 p=0,066	0,001 p=0,977	2,521 p=0,113
F											0,078 p=0,78	9,263 p<0,001
HER2												2,391 p=0,122
PD-L1												

Note – *For clarity, columns and rows are labeled as follows: AGE – age, GEN – sex, STAGE – prevalence, Lauren1 – histotype according to Lauren classification before annotation, Grade1 – histotype by degree of differentiation before annotation, Lauren2 – histotype according to Lauren classification after annotation (taking into account 70% HG), Grade2 – histotype by degree of differentiation after annotation (taking into account 70% HG), GIN-L/H – presence of dysplasia, IM – presence of intestinal metaplasia, F – presence of ulceration, HER2 – HER-2 status, PD-L1 – PD-L1 status*

Table S26 – Combination of adenocarcinomas with low- and high-grade glandular intraepithelial neoplasia

% area of dysplasia in biopsy	Number of ACs that can be combined with GINL	Number of ACs that combine with GINH	Number of ACs combined with GINL_and / or_GINH
>50	6	7	15
10:49	19	12	25
1-9	23	20	34
0	424	433	398

Table S27 – Combination of adenocarcinoma histotypes (after annotation) with low- and high-grade glandular intraepithelial neoplasia, % of carcinomas de novo

Histotype after annotation	% area of dysplasia in biopsy (GINL and / or GINH)					% of de novo carcinomas
	0% (cancer) de novo)	1-9%	10-49%	>50%	Total	
TAC-LG	100	10	19	14	143	70
TAC-HG	67	2	6	4	79	85
PAC-LG	16	1	1	-	18	89
PCC	107	2	4	6	119	90
Mixed	19	-	2	-	21	90
MAC	14	-	1	-	15	93
PCC-NOS	63	-	1	1	65	97
NDC	4	-	-	-	4	100
NED	8	-	-	-	8	100
Total	398	15	34	25	472	84

Table S28 – Combination of tumor association with dysplasia and with intestinal metaplasia

Intestinal metaplasia	<1% dysplasia in biopsy (GINL and / or GINH)	>1% dysplasia in biopsy (GINL and / or GINH)	Total
Absence	341	42	383
Presence	57	32	82
Total	398	74	472

Table S29 – Ratio of frequency of occurrence with intestinal metaplasia and with histological type according to annotation data (according to Lauren classification)

Intestinal metaplasia	Gender		Histological type according to Lauren			Total
	F	M	Intestinal type	Indeterminate type	Diffuse type	
Absence	207	176	125	85	173	383
Presence	25	64	39	28	22	89
Total	232	240	164	113	195	472

Table S30 – Combination of adenocarcinoma histotypes (after annotation by 70% HG) with intestinal metaplasia

Histotype after annotation	Absence of intestinal metaplasia	Presence of intestinal metaplasia	Total	% of carcinomas associated with intestinal metaplasia
TAC-LG	103	40	143	28
TAC-HG	58	21	79	27
PAC-LG	15	3	18	17
Mixed	18	3	21	14
PCC	102	17	119	14
PCC-NOS	60	5	65	8
NEC	8	-	8	0
MAC	15	-	15	0
NDC	4	-	4	0
Total	383	89	472	19

Figure S30 – Kaplan–Meier survival curves for low- and high-risk groups

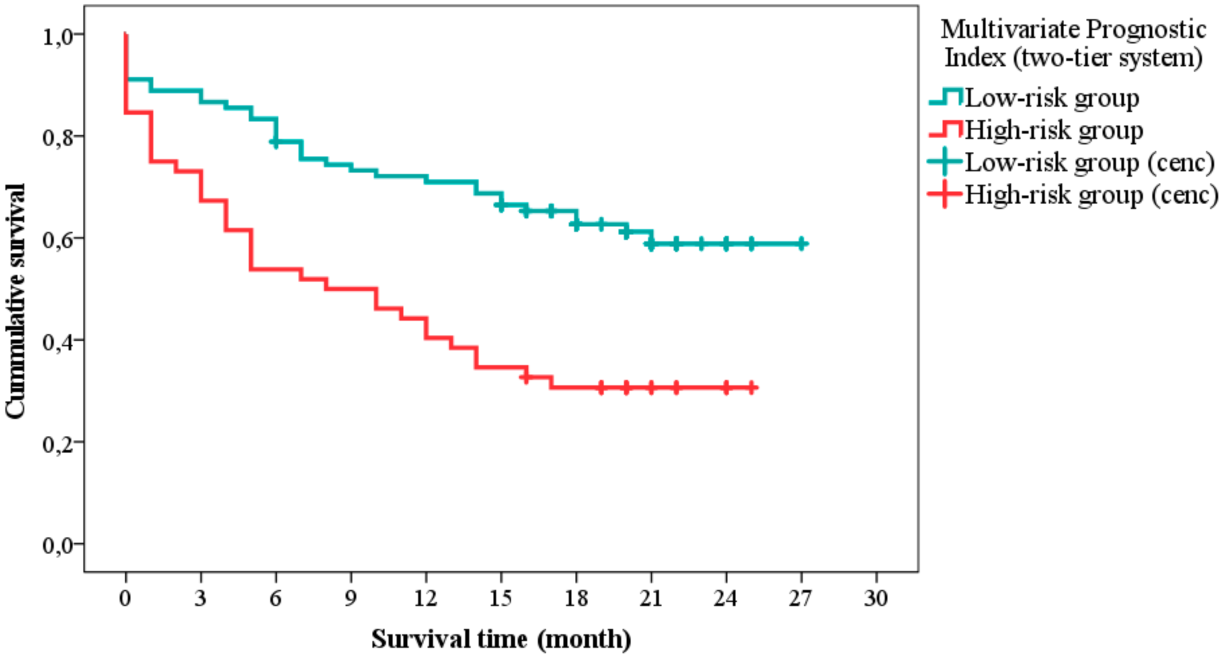

Figure S31 – Kaplan–Meier survival curves for low, intermediate and high-risk groups

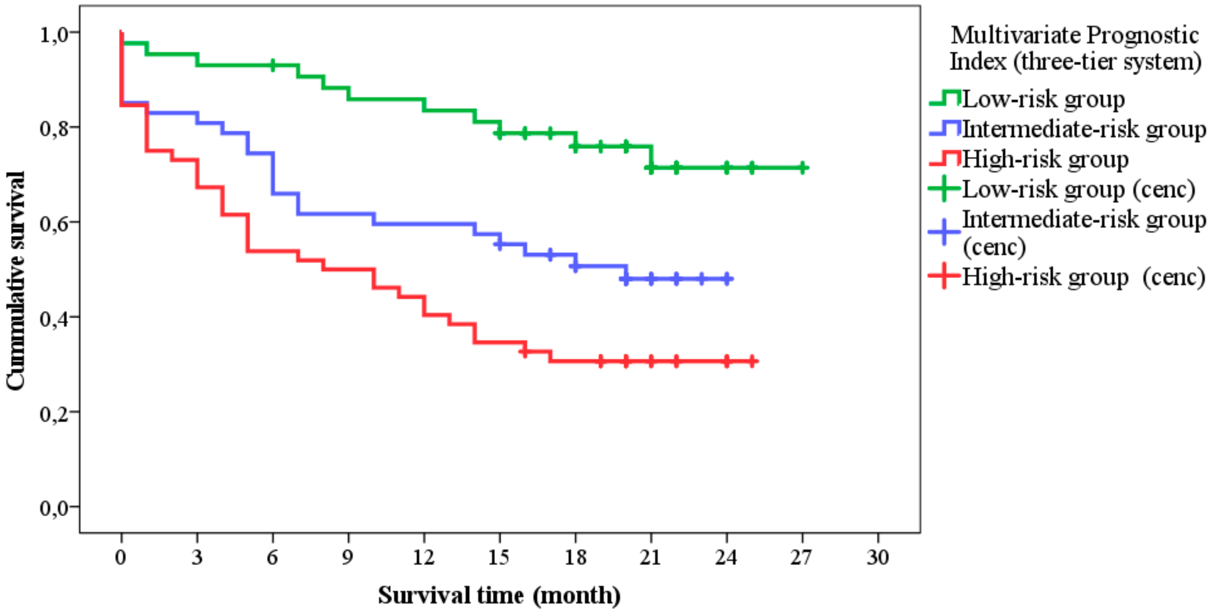

Table S31 – Comparison of different approaches to classification by histological type and options for assessing prognostic risk groups with overall survival (OS)

**Clinical and pathological parameters**	**Total n=472**	**Group with OS n = 142**	**Median survival time (months, 95% CI)**	**1-year survival (%)**	**Log - rank**	**p -value**
**Lauren histotype (primary study, before annotation)**						
Intestinal	203	60	18.0 (15.6 - 20.4)	73	5,907	0.052
Indeterminate	154	32	13.6 (10.8 - 16.4)	50		
Diffuse	110	50	14.9 (10.8 - 19.0)	50		
Mixed	5	0				
**Grade (primary research, before annotation)**						
Low-Grade	19 2	59	18.0 (15.6 - 20.4)	**74**	**5,674**	**0.017**
High-Grade	270	80	14.6 (12.1 - 17.0)	**50**		
Not specified	10	3				
**Histotype according to Lauren (after annotation according to the WHO classification, using cut-off threshold 1% HG)**						
Intestinal	113	36	17.0 (14.1 - 19.9)	75	1,711	0.425
Indeterminate	151	53	16.7 (13.8 - 19.5)	60		
Diffuse	185	47	13.8 (10.8 - 16.9)	49		
Mixed	23	6	14.0 (8.3 - 19.6)	52		
**Grade (after annotation according to the WHO classification using cut-off threshold 1% HG)**						
Low-Grade	113	39	17.3 (14.4 - 20.3)	74	0.751	0.386
High-Grade	359	103	15.8 (13.6 - 18.0)	54		
**Histotype according to Lauren (after annotation using cut-off threshold 70% of the HG)**						
Intestinal	164	62	17.5 (15.1 - 20.0)	71	4,197	0.123
Indeterminate	90	35	15.2 (11.6 - 18.7)	55		
Diffuse	195	45	13.7 (10.6 - 16.8)	49		
Mixed	23	6	14.0 (8.3 - 19.6)	52		
**Grade (after annotation using cut-off threshold 70% of the HG component)**						
Low-Grade	161	62	17.6 (15.2 - 20.0)	**71**	**4,196**	**0.41**
High-Grade	311	80	14.8 (12.3 - 17.3)	**51**		
**Prognostic risk groups (2-tier system)**						
Low risk	297	90	19.1 (17.0 - 21.3)	**71**	**14,751**	**<0.001**
High risk	175	52	11.3 (8.6 - 14.1)	**40**		
**Prognostic risk groups (3-tier system)**						
Low risk	142	43	22.3 (19.8 - 24.9)	**84**	**19,570**	**<0.001**
Risk gap	155	47	14.8 (12.0 - 17.7)	**60**		
High risk	175	52	11.3 (8.6 - 14.1)	**40**		

Note : *CI – confidence interval, OS – overall survival*

Table S32 – Univariate survival analysis of low- and high-risk groups stratified by prevalence level

Prevalence	Median survival time (months, 95% CI)		Log-rank	p -value
	Low risk	High risk		
Localized	24.6 (22.0 – 27.2)	22.1 (18.7 – 25.1)		
Regional	20.0 (16.1 – 23.9)	14.7 (9.2 – 20.1)	42,914	<0.0001
Generalized	13.1 (10.6 – 15.6)	8.4 (5.5 – 11.3)		

Figure S32 – Correlation matrix of clinicopathological parameters with the addition **of a prognostic index** ; cell values are presented in the following format: Pearson Chi-square coefficient, statistical significance (p).

	AGE	GEN	STAGE	Lauren1	Grade1	Lauren2	Grade2	GIN-L/H	IM	F	**INDEX**	HER2	PD-L1
AGE		0,947 p=0,33	1,327 p=0,51	0,361 p=0,835	0,053 p=0,819	3,625 p=0,163	0,199 p=0,656	1,031 p=0,31	0,091 p=0,763	0,445 p=0,505	25,717 p<0,001	0,952 p=0,329	1,523 p=0,218
GEN			5,048 p=0,08	1,132 p=0,568	1,125 p=0,289	0,282 p=0,869	0,154 p=0,695	0,167 p=0,683	9,567 p<0,001	1,099 p=0,295	0,545 p=0,761	2,914 p=0,088	0,026 p=0,871
STAGE				10,323 p=0,035	8,621 p=0,013	9,055 p=0,06	6,606 p=0,037	5,264 p=0,072	4,927 p=0,085	2,591 p=0,274	9,083 p=0,059	2,058 p=0,357	8,373 p=0,015
Lauren1					157,313 p<0,001	151,199 p<0,001	98,649 p<0,001	7,325 p=0,026	2,874 p=0,238	2,286 p=0,319	40,81 p<0,001	5,164 p=0,076	8,225 p=0,016
Grade1						96,309 p<0,001	95,691 p<0,001	4,906 p=0,027	1,521 p=0,218	0,748 p=0,387	36,522 p<0,001	3,934 p=0,047	0,086 p=0,77
Lauren2							458,896 p<0,001	27,491 p<0,001	12,051 p<0,001	3,521 p=0,171	39,472 p<0,001	7,692 p=0,021	0,451 p=0,798
Grade2								27,838 p<0,001	4,601 p=0,032	0,436 p=0,509	25,093 p<0,001	5,466 p=0,019	0,399 p=0,528
GIN-L/H									34,113 p<0,001	0,163 p=0,687	48,859 p<0,001	0,262 p=0,609	0,402 p=0,526
IM										3,389 p=0,066	40,139 p<0,001	0,001 p=0,977	2,521 p=0,113
F											20,279 p<0,001	0,078 p=0,78	9,263 p<0,001
**INDEX**												3,921 p=0,141	1,350 p=0,509
HER2													2,391 p=0,122
PD-L1													

Note – *For clarity, columns and rows are labeled as follows: AGE – age, GEN – gender, STAGE – prevalence, Lauren1 – histotype according to the Lauren classification before annotation, Grade1 – histotype by the degree of differentiation before annotation, Lauren2 –*

histotype according to the Lauren classification after annotation, Grade2 – histotype by the degree of differentiation after annotation, GIN-L/H – the presence of dysplasia, IM – the presence of intestinal metaplasia, F – the presence of ulceration, HER2 – HER-2 status, PD-L1 – PD-L1 status, INDEX – prognostic index

Table S33 – Characteristics of patients in the test sample by gender and age

ICD-10 original code	Gender		Total
	F	M	
C16.9	43	57	100
D00.2	10	15	25
D13.1	12	13	25
K29.7	76	24	100
Total	141	109	250

Table S34 – Average age of patients in the test sample

ICD-10 original code	Gender		Total
	Female	Male	
C16.9	68.3	67.5	67.9
D00.2	75.8	65.9	69.8
D13.1	70.3	63.3	66.6
K29.7	57.7	58.7	57.9
Total	63.3	64.9	64.0

Table S35 – Discrepancies between the auditor's opinion and the opinion of the doctor who gave the first opinion on the material

Primary ICD-10	ICD-10 Auditor (238 matches out of 250)				Total
	C16.9	D00.2	D13.1	K29.7	
C16.9	100	-	-	-	100
D00.2	7	14	4	-	25
D13.1	-	1	24	-	25
K29.7	-	-	-	100	100
Total	107	15	28	100	250

Table S36 – Register of cases of revision of gastric intestinal biopsies with the primary opinion, the opinion of the auditor, two experts and the decision of the council

No.	Floor	Age	Original ICD-10	ICD-10 auditor	Expert 1 ICD-10	Expert 2 ICD-10	Degree of agreement between the auditor and experts	ICD-10 Council
1	M	71	D00.2	D13.1	C16 .9	D00.2	Disagreement with 2 experts	D13.1
2	M	83	D00.2	D13.1	D00	D00.2	Disagreement with 2 experts	D00.2
3	AND	89	D00.2	D13.1	C16.9	C16.9	Disagreement with 2 experts	C16.9
4	M	60	D00.2	C16.9	C16.9	D00.2	Disagreement with 1 expert	C16.9
5	AND	66	D13.1	D00	C16.9	D00.2	Disagreement with 1 expert	D00.2
6	M	59	D00.2	C16.9	D00.2	C16.9	Disagreement with 1 expert	C16.9
7	M	71	D00.2	D13.1	D00.2	D13.1	Disagreement with 1 expert	D13.1
8	M	59	D00.2	C16.9	C16.9	C16.9	Consent of the auditor and experts	C16.9
9	M	82	D00.2	C16.9	C16.9	C16.9	Consent of the auditor and experts	C16.9

10	M	48	D00.2	C16.9	C16.9	C16.9	Consent of the auditor and experts	C16.9
11	AND	71	D00.2	C16.9	C16.9	C16.9	Consent of the auditor and experts	C16.9
12	AND	89	D00.2	C16.9	C16.9	C16.9	Consent of the auditor and experts	C16.9

Table S37 – Discrepancies between the final opinion (auditor/experts/consultation) and the initial opinion of the physician

Primary ICD-10	ICD-10 Final (239 matches out of 250)				Total
	C16.9	D00.2	D13.1	K29.7	
C16.9	100	-	-	-	100
D00.2	8	15	2	-	25
D13.1	-	1	24	-	25
K29.7	-	-	-	100	100
Total	108	16	26	100	250

Figure S33 – Results of clarification of histological types by doctors of the expert group

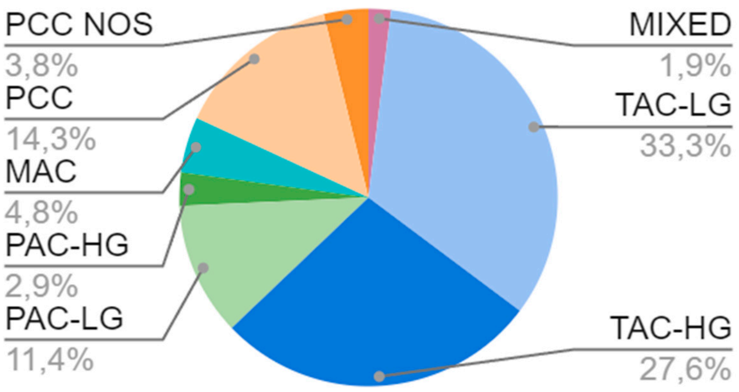

Table S38 – Registry of patients in whom discrepancies in the morphological diagnosis of gastric biopsy were identified during quality control

No.	Gender	Age	Original ICD-10	Original ICD-O	Final ICD-10	Final ICD-O	Clinical data	Observation (months)
1	M	71	D00.2	GINH	D13 .1	GINL	Submucosal resection – D00.2.	30
2	F	89	D00.2	GINH	D13 .1	GINL	Submucosal resection – D13.1 GINL.	22
3	M	60	D00.2	GINH	C16 .9	TACG1	Gastric resection – C16.9. PACG1.pT1aN0	15
4	F	66	D13.1	GINL	D00 .2	GINH	Submucosal resection – D13.1 GINL.	24
5	M	59	D00.2	GINH	C16 .9	TACG1	Submucosal resection – C16.9 TACG1.pT1a	23
6	M	71	D00.2	GINH	D13 .1	GINL	Submucosal resection – D13.1 GINL.	23
7	M	59	D00.2	GINH	C16.9	TACG1	Gastric resection – C16.9. TACG1.pT1aN0	27
8	M	82	D00.2	GINH	C16.9	PACG1	Repeat biopsy – C16.9 PACG1	19
9	M	48	D00.2	GINH	C16 .9	TACG1	Submucosal resection – C16.9. TACG1.pT1a	15
10	F	71	D00.2	GINH	C16 .9	TACG1	Repeat biopsy – C16.9 TACG1	15
11	F	89	D00.2	GINH	C16 .9	TACG1	Submucosal resection – C16.9 TACG1.pT1a	15

Table S39 – Change in diagnosis by ICD-10 code by the number of cases between the initial opinion, the opinion of the AI algorithm and the opinion of the expert group

	Opinion of the expert group		
Original opinion (below)	Non-C16.9	C16.9	Total
Non-C16.9	142	8	150
C16.9	0	100	100
Total	142	108	250

	Opinion of the expert group		
Original opinion (below)	D13.1+K29.7	C16.9+D00.2	Total
D13.1+K29.7	124	1	125
C16.9+D00.2	2	123	125
Total	126	124	250

	AI's Opinion		
Original opinion (below)	Non-C16.9	C16.9	Total
Non-C16.9	123	27	150
C16.9	26	74	100
Total	149	101	250

	AI's Opinion		
Original opinion (below)	D13.1+K29.7	C16.9+D00.2	Total
D13.1+K29.7	96	29	125
C16.9+D00.2	17	108	125
Total	113	137	250

	AI's Opinion		
Expert group (below)	Non-C16.9	C16.9	Total
Non-C16.9	120	22	142
C16.9	29	79	108
Total	149	101	250

	AI's Opinion		
Expert group (below)	D13.1+K29.7	C16.9+D00.2	Total
D13.1+K29.7	98	28	126
C16.9+D00.2	15	109	124
Total	113	137	250
